# Supplementary material for: Antioxidant and Antitumor Activities of Newly Synthesized Hesperetin Derivatives
Source: Molecules. 2022 Jan 27;27(3):879. doi: 10.3390/molecules27030879 (PMC8839103; doi:10.3390/molecules27030879)

Supplementary Materials

Antioxidant and Antitumor Activities of Newly Synthesized Hesperetin Derivatives

Guanlin Zhong, Jiayi Shen *, Zhengwang Chen, Zunxian Lin, Lipeng Long, Jiaying Wu, Chenhuan Long, Siyu Huang, Ping Lian and Guotian Luo *

Key Laboratory of Organo-Pharmaceutical Chemistry of Jiangxi Province, Gannan Normal University, Ganzhou 341000, China; z01234560327@163.com (G.Z.); chenzwang@126.com (Z.C.); linzunxiangnnu@126.com (Z.L.); long_lipeng@126.com (L.L.); realwujiaying@163.com (J.W.); LCH1809105833@163.com (C.L.); JRL336699@163.com (S.H.); lianping@gnnu.edu.cn (P.L.)

* Correspondence: 18626921895@163.com (J.S.); luoguotian@gnnu.edu.cn (G.L.); Tel./Fax: +86-797-8393670 (G.L.)

Contents

A. General method 2

B. General procedure for the synthesis of hesperetin derivatives 3

C. Analytical data 4

D. HRMS of Compounds 8

E. Antioxidant Activities Research 20

F. Anti-tumor activity assays 21

G. ^{1}H and ^{13}C NMR Spectra of Compounds 24

A. General method

All reagents and solvents were purchased and used without further purification unless specifically mentioned. The ^{1}H NMR and ^{13}C NMR spectra were obtained in $CDCl_3$ on a 400 and 100 MHz NMR spectrometer. The chemical shifts are referenced to signals at 7.26 and 77.0 ppm, respectively, and chloroform was used as the solvent with TMS as the internal standard unless otherwise noted. Silicone oil baths were used for heating. Silica gel (300-400 mesh) was used for flash column chromatograph, eluting (unless otherwise stated) with ethyl acetate/petroleum ether (PE) (60-90 ℃) mixture. HRMS data were collected from a high resolution mass spectrometer (QTOF-LCMS-9030, SHIMADZU). Usage condition : sample concentration should be diluted enough times, sample should be completely dissolved, flammable and explosive samples must be noted. Provide sample structure, molecular weight or functional groups to the extent possible in order to select ionization mode. the instrumental parameters used for mass spectrometry

Item	Setting	Units
Total Flow	0.4000	mL/min
OvenTemperature	40	C
Nebulizing Gas Flow	3.0	L/min
Drying Gas Flow	10.0	L/min
Heating Gas Flow	10.0	L/min
Interface Voltage	-3.00	kV
Interface Temperature	300	degC
Desolvation Temperature	526	degC
DL Temperature	250	degC
Heat Block Temperature	400	degC
Detector Voltage	1.98	kV
CID Gas	230	kPa
Sub-nebulizing	1.0	L/min

TLC was used on commercially available 100−400 mesh silica gel plates (GF254).

B. General procedure for the synthesis of hesperetin derivatives

(1) Synthesis of hesperetin **2**

Take hesperidin 1 about 3.5 g. 5.73 mmol, adding methanol 280 ml, adding 10 ml 96% H_2SO_4 while stirring. The reaction solution was set heating temperature 70 °C and refluxing 7.5 h. After the reaction stopped heating, the reaction mixture was cooled to room temperature. Then the reaction solution was extracted and poured into EtOAc about 300 mL. The organic phase was washed with water (3 × 400 ml) until the lower aqueous phase was colorless and transparent, and then the upper organic phase was concentrated. Then acetone was used to dissolve the residue about 70 mL and drop it into the hot 1 % AcOH aqueous solution about 700 mL. The heating temperature was set at 70 °C and the stirring was 1.5. Solution cooling, filtration, drying. Hesperetin was obtained.

(2) Synthesis of hesperetin derivatives (**3a**-**3k**)

0.604g of hesperetin (2 mmol) was taken in the reaction vessel, 40 ml of methanol was added to dissolve it, then added 204 μL of benzaldehyde (2 mmo1, 1.0 equivalent) and 1.1 equivalent of the amine-containing compound, the reaction temperature was at 40 ℃. The reaction was completed after stirring for 8h, then it reaction was monitored by TLC. The reaction mixture is concentrated and evaporated in vacuum to obtain crude products. The reaction mixture was concentrated and evaporated in vacuum, and extracted with ethyl acetate-water to a colorless and transparent aqueous phase. The organic layer of ethyl acetate was retained and dried with anhydrous Na_2SO_4 for 4 h. The mixture was concentrated under reduced pressure, get rough stuff and the rough stuff was purified by silica gel column chromatography (EtOAc /PE, 3:1→1:1) to

obtain compounds (3**a**-3**k**).

C. Analytical data

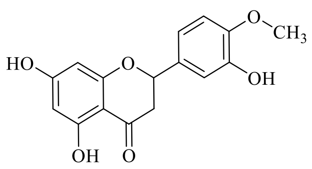

5,7-dihydroxy-2-(3-hydroxy-4-methoxyphenyl)chroman-4-one hesperetin (**2**)

Yellow solid, 85% yield, ^{1}H NMR (400 MHz, DMSO-*d6*) δ = 12.12 (s, 1H, 5-OH), 10.78 (s, 1H, 7-OH), 9.08 (s, 1H, 3'-OH), 7.06 – 6.81 (m, 3H, 2'-H, 5'-H, 6'-H), 5.89 (dd, *J*=5.7, 2.1, 2H, 8-H, 6-H), 5.43 (dd, *J*=12.3, 3.1, 1H, 2-H), 3.78 (s, 3H, OCH_3), 3.19 (dd, *J*=17.1, 12.3, 1H, 3-H), 2.71 (dd, *J*=17.1, 3.2, 1H, 3-H). ^{13}C NMR (100 MHz, DMSO-*d6*) δ = 196.36, 166.77, 163.60, 162.93, 148.01, 146.57, 131.25, 117.81, 114.18, 112.05, 101.93, 95.93, 95.12, 78.36, 55.77, 42.19. HRMS : m/z [M-H]$^-$ calcd for. $C_{16}H_{13}O_6$: 301.07176, found: 301.07180.

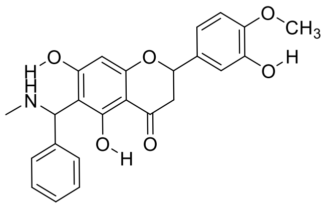

5,7-dihydroxy-2-(3-hydroxy-4-methoxyphenyl)-6-((methylamino)(phenyl)methyl)chroman-4-one (**3a**)

Yellow solid, 55% yield, ^{1}H NMR (400 MHz, $CDCl_3$) δ = 12.48 (s, 1H, OH), 7.56 – 7.26 (m, 5H, Ar-H), 7.00 (dd, *J*=3.7, 2.0, 1H, 5'-H), 6.88 (ddd, *J*=17.3, 9.7, 5.1, 2H, 2'-H, 6'-H), 6.00 (dd, *J*=11.3, 4.9, 1H, 8-H), 5.39 – 5.01 (m, 2H, N-CH-Ar, 2-H), 4.00 – 3.72 (m, 3H, OCH_3), 2.98 (ddd, *J*=18.7, 17.2, 13.0, 1H, 3-H), 2.68 (dd, *J*=17.1, 3.0, 1H, 3-H), 2.55 (dd, *J*=12.4, 6.3, 3H, N-CH_3). ^{13}C NMR (100 MHz, $CDCl_3$) δ = 195.43, 168.80, 162.26, 161.55, 161.49, 146.90, 145.81, 140.89, 131.67, 128.89, 128.14, 127.15, 118.13, 118.09, 112.66, 110.64, 103.52, 101.81, 96.63, 96.57, 78.71, 62.20, 55.99, 43.12, 42.91, 34.43. HRMS : m/z [M-H]$^-$ calcd for. $C_{24}H_{23}NO_6$: 420.14526, found: 420.14510.

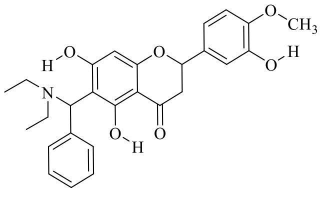

6-((diethylamino)(phenyl)methyl)-5,7-dihydroxy-2-(3-hydroxy-4-methoxyphenyl)chroman-4-one (**3b**)

Yellow solid, 60% yield, ^{1}H NMR (400 MHz, DMSO-*d6*) δ = 12.72 (s, 1H, 5-OH), 9.08 (s, 1H, 3'-H), 7.53 (d, *J*=7.1, 2H, Ar-H), 7.32 (ddd, *J*=15.6, 7.4, 3.4, 3H, Ar-H), 6.93 – 6.87 (m, 2H, 6'-H, 2'-H), 6.83 (d, *J*=8.3, 1H, 5'-H), 5.73 (d, *J*=2.1, 1H, 8-H), 5.37 – 5.30 (m, 1H, 2-H), 5.09 (d, *J*=8.4, 1H, N-CH-Ar), 3.76 (d, *J*=2.2, 3H, OCH_3), 3.09 (dd, *J*=17.7, 5.3, 1H, 3-H), 2.75 (dd, *J*=14.5, 7.1, 4H, N-CH_2), 2.57 (dd, *J*=17.4, 2.9, 1H, 3-H), 1.00 (t, *J*=6.9, 6H, CH_3). ^{13}C NMR (100 MHz, $CDCl_3$) δ = 194.41, 171.70, 162.20, 160.71, 147.23, 146.12, 138.04, 131.39, 128.57,

128.09, 117.67, 112.84, 110.70, 104.69, 100.45, 97.22, 78.50, 64.93, 55.65, 42.70, 42.55, 13.93. HRMS : m/z [M-H]$^-$ calcd for. $C_{27}H_{29}NO_6$: 462.19221, found: 462.19215.

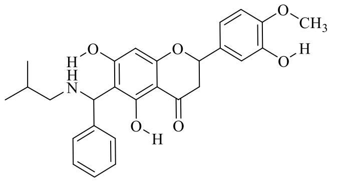

5,7-dihydroxy-2-(3-hydroxy-4-methoxyphenyl)-6-((isobutylamino)(phenyl)methyl)chroman-4-one (**3c**)

Yellow solid, 50% yield, ^{1}H NMR (400 MHz, $CDCl_3$) δ = 12.45 (s, 1H, OH), 7.53 – 7.43 (m, 2H, Ar-H), 7.36 – 7.26 (m, 3H, Ar-H), 7.02 (dd, *J*=4.0, 2.0, 1H, 6'-H), 6.90 (ddd, *J*=17.2, 9.6, 5.1, 2H, 2'-H, 5'-H), 6.00 (d, *J*=2.6, 1H, 8-H), 5.32 – 5.21 (m, 2H, N-CH-Ar, 2-H), 3.90 (s, 3H, OCH_3), 2.98 (dt, *J*=13.2, 4.1, 1H, 3-H), 2.75 – 2.66 (m, 1H, 3-H), 2.66 – 2.54 (m, 2H, CH_2), 1.86 (td, *J*=13.4, 6.7, 1H, CH), 1.01 – 0.91 (m, 6H, CH_3). ^{13}C NMR (100 MHz, DMSO-*d6*) δ = 194.22, 173.50, 161.80, 160.72, 147.90, 146.55, 140.34, 131.67, 128.60, 127.93, 127.60, 117.65, 114.08, 112.06, 103.23, 96.98, 77.99, 77.86, 55.75, 55.00, 54.55, 41.90, 26.54, 20.29. HRMS : m/z [M-H]$^-$ calcd for. $C_{27}H_{29}NO_6$: 462.19221, found: 462.19247.

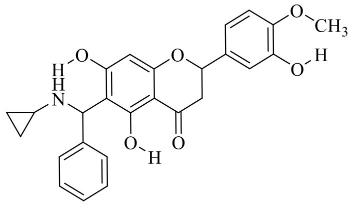

6-((cyclopropylamino)(phenyl)methyl)-5,7-dihydroxy-2-(3-hydroxy-4-methoxyphenyl)chroman-4-one (**3d**)

Yellow solid, 61% yield, ^{1}H NMR (400 MHz, $CDCl_3$) δ = 12.45 (s, 1H, OH), 7.47 – 7.38 (m, 2H, Ar-H), 7.34 – 7.21 (m, 3H, Ar-H), 7.02 (dd, *J*=3.9, 2.0, 1H, 6'-H), 6.89 (dd, *J*=19.1, 8.3, 2H, 2'-H, 5'-H), 6.00 (d, *J*=1.5, 1H, 8-H), 5.45 (d, *J*=9.3, 1H, 2-H), 5.31 – 5.22 (m, 1H, N-CH-Ar), 3.90 (s, 3H, OCH_3), 3.01 (ddd, *J*=20.6, 17.1, 13.1, 1H, 3-H), 2.70 (dt, *J*=17.1, 3.0, 1H, 3-H), 2.39 (tdd, *J*=6.9, 3.9, 1.9, 1H, CH), 0.67 – 0.37 (m, 4H, CH_2). ^{13}C NMR (100 MHz, $CDCl_3$) δ = 195.64, 168.13, 162.05, 161.13, 146.90, 145.85, 141.79, 131.76, 128.90, 127.96, 127.13, 127.11, 118.13, 112.67, 112.62, 110.64, 105.22, 105.14, 102.10, 96.58, 96.50, 78.84, 61.17, 56.02, 43.23, 31.08, 5.57. HRMS : m/z [M-H]$^-$ calcd for. $C_{26}H_{25}NO_6$: 446.16091, found: 446.16038.

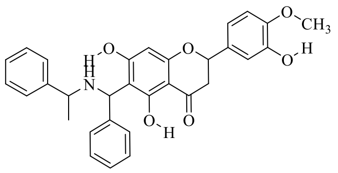

5,7-dihydroxy-2-(3-hydroxy-4-methoxyphenyl)-6-(phenyl((1-phenylethyl)amino)methyl)chroman-4-one (**3e**)

Yellow solid, 55% yield, ^{1}H NMR (400 MHz, $CDCl_3$) δ = 12.31 (s, 1H, OH), 7.45 – 7.27 (m, 4H, Ar-H), 7.25 – 7.18 (m, 6H, Ar-H), 7.00 (td, *J*=5.0, 2.0, 1H, 6'-H), 6.91 – 6.80 (m, 2H, 2'-H, 5'-H), 6.00 (d, *J*=47.5, 1H, 8-H), 5.33 – 5.17 (m, 1H, 2-H), 5.12 (d, *J*=11.3, 1H, N-CH-Ar), 3.90 (dd, *J*=22.5, 6.7, 1H, NH), 3.83 (dd, *J*=3.4, 1.4, 3H, OCH_3), 2.97 (ddd, *J*=26.1, 17.1, 13.2, 1H, 3-H), 2.69 – 2.60 (m, 1H, 3-H), 1.57 (dd, *J*=6.7, 2.4, 1H, CH_3), 1.46 (d, *J*=6.8, 2H, CH_3). ^{13}C NMR (100 MHz, $CDCl_3$) δ =195.47 , 162.11 , 161.29 , 160.80 , 146.93 , 145.80 , 142.25 , 141.49 , 131.55 , 128.84 , 128.57 , 127.91 , 127.77 , 127.12 , 126.44 , 117.98 , 112.71 , 110.66 , 103.73 , 101.79 , 96.54 , 78.54 , 60.35 , 55.85 , 43.37 , 29.56 . HRMS : m/z [M-H]$^-$

calcd for. $C_{31}H_{29}NO_6$: 510.19221, found: 510.19256.

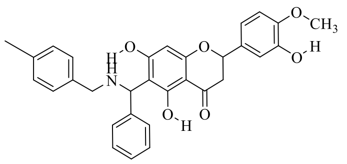

5,7-dihydroxy-2-(3-hydroxy-4-methoxyphenyl)-6-(((4-methylbenzyl)amino)(phenyl)methyl)chroman-4-one(**3f**)

Yellow solid, 47% yield, ^{1}H NMR (400 MHz, $CDCl_3$) δ = 12.48 (s, 1H, OH), 7.41 (d, *J*=8.0, 2H, Ar-H), 7.32 – 7.26 (m, 2H, Ar-H), 7.24 – 7.13 (m, 5H, Ar-H), 7.01 (dd, *J*=4.3, 2.0, 1H, Ar-H), 6.88 (dt, *J*=18.5, 7.4, 2H, 2'-H, 5'-H), 6.04 (s, 1H, 8-H), 5.41 (d, *J*=9.5, 1H, N-CH-Ar), 5.25 (td, *J*=14.3, 2.9, 1H, 2-H), 3.92 (t, *J*=7.9, 1H, N-CH_2), 3.87 (s, 3H, OCH_3), 3.77 (dd, *J*=12.8, 2.9, 1H, N-CH_2), 3.00 (ddd, *J*=22.7, 17.1, 13.0, 1H, 3-H), 2.69 (dd, *J*=17.1, 2.9, 1H, 3-H), 2.33 (s, 3H, CH_3). ^{13}C NMR (100 MHz, $CDCl_3$) δ = 195.55, 168.50, 162.14, 161.50, 146.91, 145.83, 141.55, 137.57, 134.31, 131.66, 129.44, 128.82, 128.50, 127.92, 127.05, 118.08, 112.66, 110.64, 103.95, 102.02, 96.52, 78.72, 59.45, 55.94, 51.97, 42.92, 21.06. HRMS : m/z [M-H]$^-$ calcd for. $C_{31}H_{29}NO_6$: 510.19221, found: 510.19277.

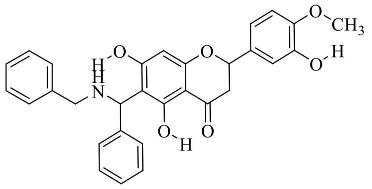

6-((benzylamino)(phenyl)methyl)-5,7-dihydroxy-2-(3-hydroxy-4-methoxyphenyl)chroman-4-one (**3g**)

Yellow solid, 66% yield, ^{1}H NMR (400 MHz, $CDCl_3$) δ = 12.46 (s, 1H, OH), 7.40 (d, *J*=7.8, 2H, Ar-H), 7.32 – 7.26 (m, 3H, Ar-H), 7.26 – 7.13 (m, 5H, Ar-H), 7.00 (dd, *J*=4.1, 2.0, 1H, 6'-H), 6.86 (dt, *J*=16.3, 7.3, 2H, 2'-H, 5'-H), 5.99 (d, *J*=3.1, 1H, 8-H), 5.32 (d, *J*=8.6, 1H, N-CH-Ar), 5.26 – 5.15 (m, 1H, 2-H), 3.86 (s, 3H, OCH_3), 3.04 (dd, *J*=10.3, 4.4, 2H, N-CH_2), 2.83 (dd, *J*=14.4, 7.6, 1H, 3-H), 2.67 (dd, *J*=17.1, 3.0, 1H, 3-H). ^{13}C NMR (100 MHz, $CDCl_3$) δ = 195.51, 168.47, 162.08, 161.28, 146.89, 145.83, 141.49, 138.33, 131.68, 128.84, 128.70, 128.60, 127.93, 127.04, 127.00, 126.62, 118.10, 112.63, 110.62, 104.26, 101.97, 96.49, 78.72, 59.92, 55.97, 49.02, 42.93. HRMS : m/z [M-H]$^-$ calcd for. $C_{30}H_{27}NO_6$: 496.17656, found: 496.17570.

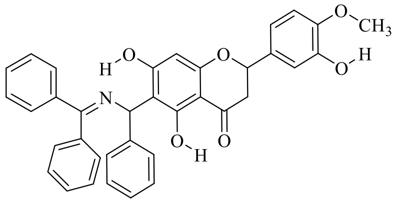

6-(((diphenylmethylene)amino)(phenyl)methyl)-5,7-dihydroxy-2-(3-hydroxy-4-methoxyphenyl)chroman-4-one (**3h**)

Yellow solid, 45% yield, ^{1}H NMR (400 MHz, $CDCl_3$) δ = 12.37 (d, *J*=11.0, 1H, OH), 7.88 – 7.25 (m, 11H, Ar-H), 7.27 – 7.04 (m, 4H, Ar-H), 6.99 (dd, *J*=4.8, 2.2, 1H, 6'-H), 6.91 – 6.76 (m, 2H, 2'-H, 5'-H), 6.08 (dd, *J*=6.2, 3.3, 1H, 8-H), 5.21 (ddd, *J*=12.6, 5.6, 2.6, 1H, 2-H), 3.79 (d, *J*=2.6, 3H, OCH_3), 3.08 – 2.86 (m, 1H, 3-H), 2.76 – 2.60 (m, 1H, 3-H). ^{13}C NMR (100 MHz, $CDCl_3$) δ = 195.67, 170.48, 167.40, 162.05, 160.16, 146.89, 145.83, 142.53, 138.32, 134.55, 131.28, 130.01, 129.54, 128.99, 128.61, 128.45, 127.94, 127.13, 126.82, 118.13, 112.63, 110.65, 110.62, 108.17, 102.09, 102.02, 96.98, 96.86, 78.72, 62.74, 55.96, 42.92. HRMS : m/z [M-H]$^-$ calcd for. $C_{36}H_{29}NO_6$: 570.19221, found: 570.19219.

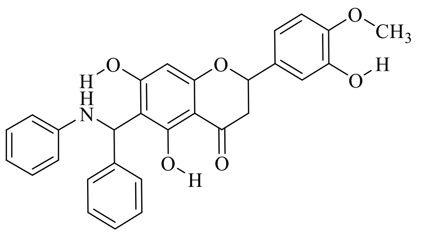

5,7-dihydroxy-2-(3-hydroxy-4-methoxyphenyl)-6-(phenyl(phenylamino)methyl)chroman-4-one (**3i**)

Yellow solid, 45% yield, ^{1}H NMR (400 MHz, $CDCl_3$) δ = 12.44 (s, 1H, OH), 7.39 (d, *J*=7.2, 2H, Ar-H), 7.27 – 7.06 (m, 5H, Ar-H), 6.93 – 6.60 (m, 6H, Ar-H, 6'-H, 2'-H, 5'-H), 5.91 (s, 1H, 8-H), 5.75 (d, *J*=9.7, 1H, 2-H), 5.14 (dd, *J*=21.1, 12.0, 1H, N-CH-Ar), 3.74 (s, 3H, OH_3), 2.90 (ddd, *J*=22.5, 17.1, 13.4, 1H, 3-H), 2.65 – 2.53 (m, 1H, 3-H). ^{13}C NMR (100 MHz, $CDCl_3$) δ = 195.91, 166.80, 161.98, 160.49, 160.46, 146.93, 146.07, 145.73, 141.24, 141.19, 131.35, 129.31, 128.97, 128.17, 127.26, 121.89, 118.04, 116.40, 112.66, 110.64, 105.27, 102.31, 96.51, 96.40, 78.55, 58.49, 55.83, 42.89. HRMS : m/z [M-H]$^-$ calcd for. $C_{29}H_{25}NO_6$: 482.16091, found: 482.16040.

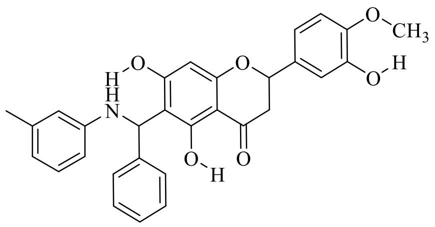

5,7-dihydroxy-2-(3-hydroxy-4-methoxyphenyl)-6-(phenyl(m-tolylamino)methyl)chroman-4-one (**3j**)

Yellow solid, 52% yield, ^{1}H NMR (400 MHz, $CDCl_3$) δ = 12.49 (s, 1H, OH), 9.48 (s, 1H, OH), 7.49 – 7.26 (m, 5H， Ar-H), 7.32 – 7.17 (m, 4H, Ar-H), 6.99 (t, *J*=2.3, 1H, 6'-H), 6.85 (dt, *J*=19.3, 7.3, 2H, 2'-H, 5'-H), 6.04 (d, *J*=3.2, 1H, 8-H), 5.83 (d, *J*=6.1, 1H, 2-H), 5.24 (ddd, *J*=12.4, 9.4, 2.9, 1H, N-CH-Ar), 3.82 (s, 3H, OCH_3), 3.47 (d, *J*=1.7, 3H, CH_3), 3.00 (ddd, *J*=18.8, 17.3, 13.0, 1H, 3-H), 2.71 (ddd, *J*=17.2, 3.0, 1.3, 1H, 3-H). ^{13}C NMR (100 MHz, $CDCl_3$) δ = 196.04, 164.93, 162.28, 160.73, 146.95, 145.80, 142.09, 140.81, 139.70, 131.37, 128.43, 128.04, 127.85, 126.73, 126.70, 126.46, 126.20, 118.07, 118.04, 112.63, 110.64, 110.62, 107.40, 105.64, 102.31, 102.24, 96.63, 80.19, 57.42, 55.91, 43.08, 20.94. HRMS: m/z [M-H]$_-$ calcd for. $C_{30}H_{27}NO_6$: 496.17656, found: 496.17571.

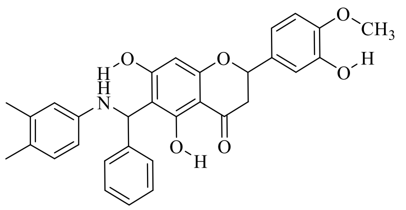

6-(((3,4-dimethylphenyl)amino)(phenyl)methyl)-5,7-dihydroxy-2-(3-hydroxy-4-methoxyphenyl)chroman-4-one (**3k**)

Yellow solid, 46% yield, ^{1}H NMR (400 MHz, $CDCl_3$) δ = 12.51 (s, 1H, OH), 7.50 – 7.43 (m, 3H, Ar-H), 7.36 – 7.28 (m, 3H, Ar-H), 7.03 – 6.96 (m, 2H, Ar-H), 6.90 (dd, *J*=8.3, 2.0, 1H, NH), 6.85 (d, *J*=8.3, 1H, 6'-H), 6.70 – 6.61 (m, 2H, 2'-H, 5'-H), 6.01 (s, 1H, 8-H), 5.79 (d, *J*=9.0, 1H, 2-H), 5.27 (ddd, *J*=21.9, 13.2, 2.9, 1H, N-CH-Ar), 3.88 (d, *J*=1.0, 3H, OCH_3), 3.02 (ddd, *J*=24.5, 17.2, 13.2, 1H, 3-H), 2.71 (ddd, *J*=17.2, 6.0, 3.0, 1H, 3-H), 2.17 (s, 6H, CH_3). ^{13}C NMR (100 MHz, $CDCl_3$) δ = 195.92, 167.33, 162.05, 160.60, 146.94, 145.83, 143.84, 141.54, 141.49, 137.62, 133.62, 131.62, 130.57, 130.46, 130.12, 129.09, 128.41, 128.25, 127.35, 118.11, 113.88, 112.68, 110.68, 105.25, 105.20, 102.32, 102.29, 96.64, 96.55, 78.87, 59.24, 55.98, 43.16, 19.95, 18.86. HRMS : m/z [M-H]$^-$ calcd for. $C_{31}H_{29}NO_6$: 510.19221, found: 510.19286.

D. HRMS of Compounds

Formula Predictor Report Printed at 2020/12/15 21:01:58

Formula Predictor Result	C16 H13 O6
Mass	301.0718
Error Margin	5 ppm
DBE Range	Not Used
Electron Ions	Both configurations
HC Ratio	Not Used
Nitrogen Rule	Not Used

#	Score	Pred. (M)	Pred. m/z	Meas. m/z	Diff. (mDa)	Formulae (M)	Ion	Diff. (ppm)	Iso Score	DBE
1	59.22	301.07121	301.07176	301.07180	0.04	C16 H13 O6	[M]-	0.133	54.73	10.5

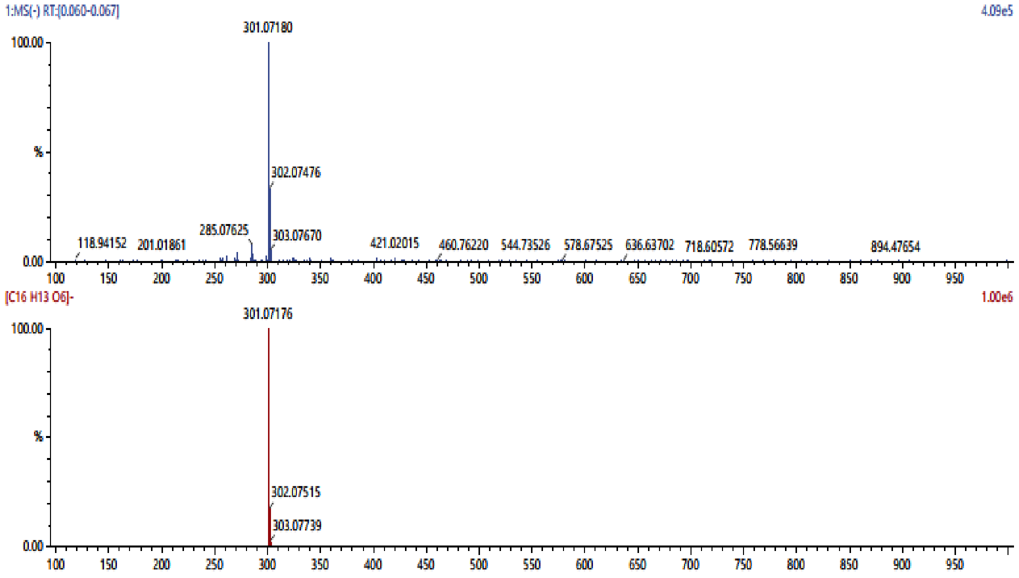

---- Page 1 of 1

HRMS of **2**

HRMS : m/z [M-H]$^-$ calcd for. $C_{16}H_{13}O_6$: 301.07176, found: 301.07180.

Formula Predictor Report

Printed at 2020/12/15 21:28:16

Formula Predictor Result	C24 H23 N O6
Mass	420.1451
Error Margin	5 ppm
DBE Range	Not Used
Electron Ions	Both configurations
HC Ratio	Not Used
Nitrogen Rule	Not Used

#	Score	Pred. (M)	Pred. m/z	Meas. m/z	Diff. (mDa)	Formulae (M)	Ion	Diff. (ppm)	Iso Score	DBE
1	99.85	421.15254	420.14526	420.14510	-0.16	C24 H23 N O6	[M-H]-	-0.381	100.00	14.0

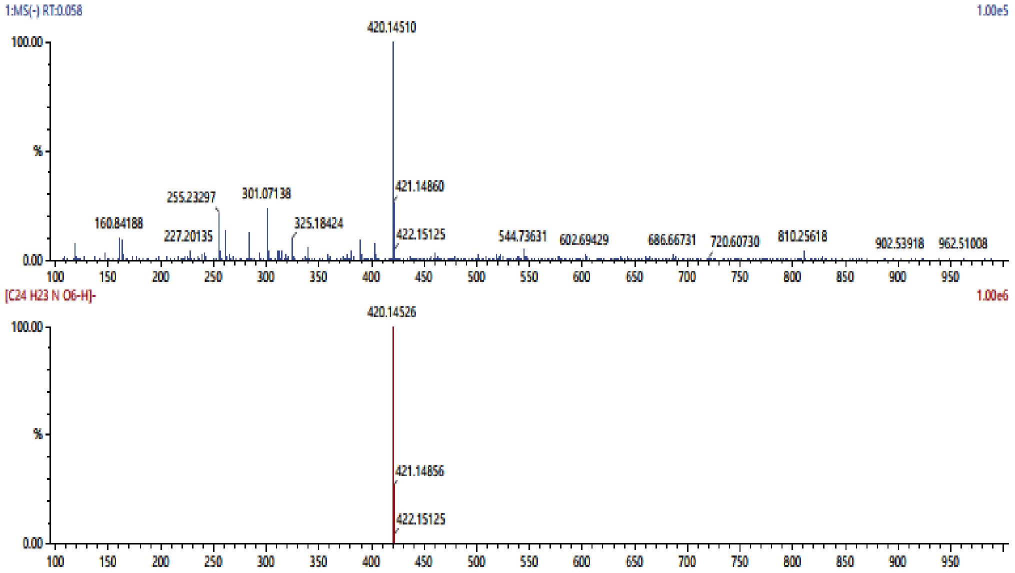

HRMS of **3a**

HRMS : m/z [M-H]$^-$ calcd for. $C_{24}H_{23}NO_6$: 420.14526, found: 420.14510.

Formula Predictor Report

Printed at 2020/12/15 21:14:24

Formula Predictor Result	C27 H29 N O6
Mass	462.19215
Error Margin	5 ppm
DBE Range	Not Used
Electron Ions	Both configurations
HC Ratio	Not Used
Nitrogen Rule	Not Used

#	Score	Pred. (M)	Pred. m/z	Meas. m/z	Diff. (mDa)	Formulae (M)	Ion	Diff. (ppm)	Iso Score	DBE
4	86.85	463.19949	462.19221	462.19215	-0.06	C27 H29 N O6	[M-H]-	-0.130	85.43	14.0

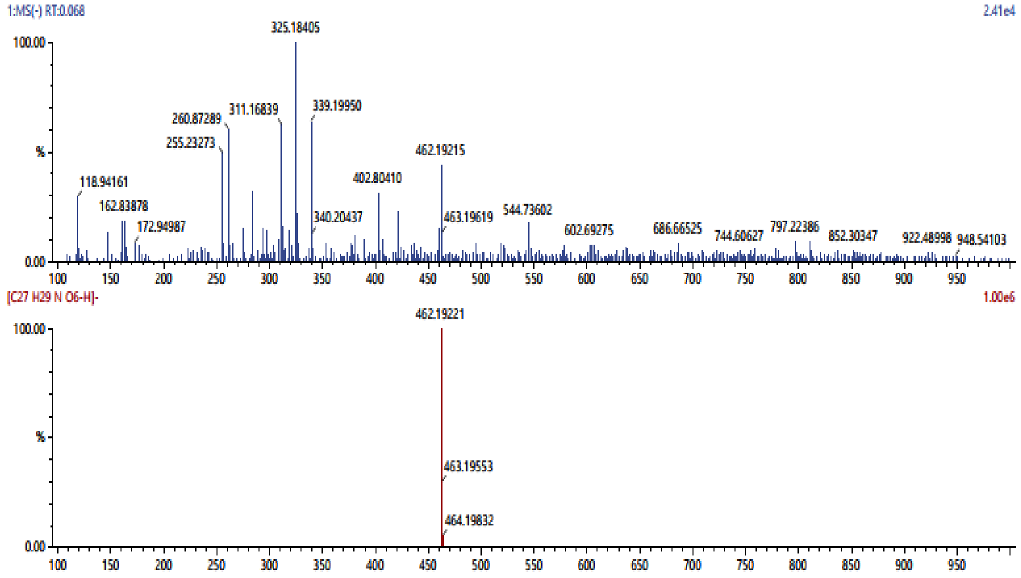

HRMS of **3b**

HRMS : m/z [M-H]$^-$ calcd for. $C_{27}H_{29}NO_6$: 462.19221, found: 462.19215.

Formula Predictor Report Printed at 2020/12/15 21:30:03

Formula Predictor Result	C27 H29 N O6
Mass	462.19247
Error Margin	5 ppm
DBE Range	Not Used
Electron Ions	Both configurations
HC Ratio	Not Used
Nitrogen Rule	Not Used

#	Score	Pred. (M)	Pred. m/z	Meas. m/z	Diff. (mDa)	Formulae (M)	Ion	Diff. (ppm)	Iso Score	DBE
1	99.59	463.19949	462.19221	462.19247	0.26	C27 H29 N O6	[M-H]-	0.563	99.81	14.0

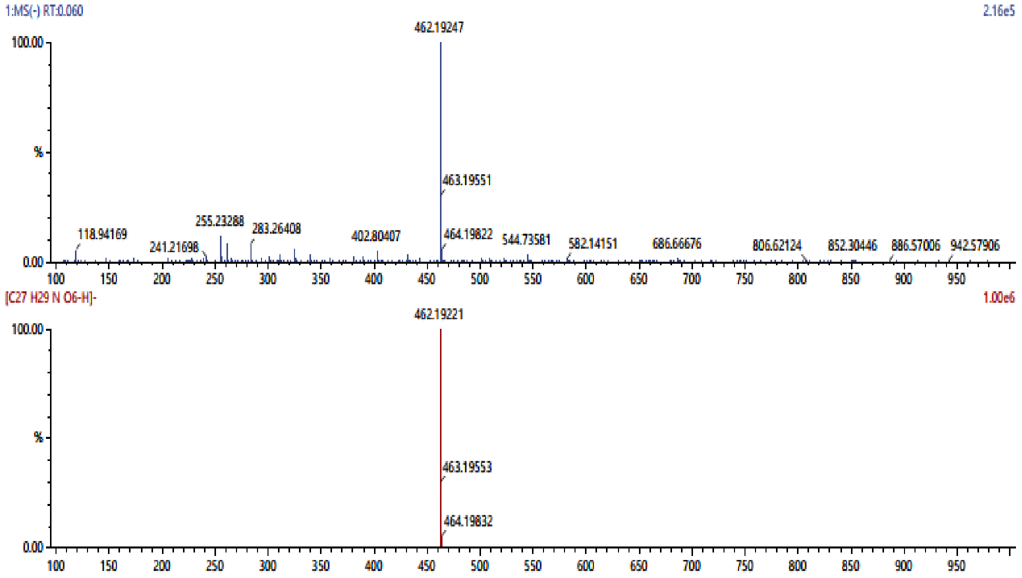

**** Page 1 of 1

HRMS of **3c**

HRMS : m/z [M-H]$^-$ calcd for. $C_{27}H_{29}NO_6$: 462.19221, found: 462.19247.

Formula Predictor Report

Printed at 2021/1/12 20:57:35

Formula Predictor Result	C26 H25 N O6
Mass	446.16038
Error Margin	5 ppm
DBE Range	Not Used
Electron Ions	Both configurations
HC Ratio	Not Used
Nitrogen Rule	Not Used

#	Score	Pred. (M)	Pred. m/z	Meas. m/z	Diff. (mDa)	Formulae (M)	Ion	Diff. (ppm)	Iso Score	DBE
1	96.29	447.16819	446.16091	446.16038	-0.53	C26 H25 N O6	[M-H]-	-1.188	96.50	15.0

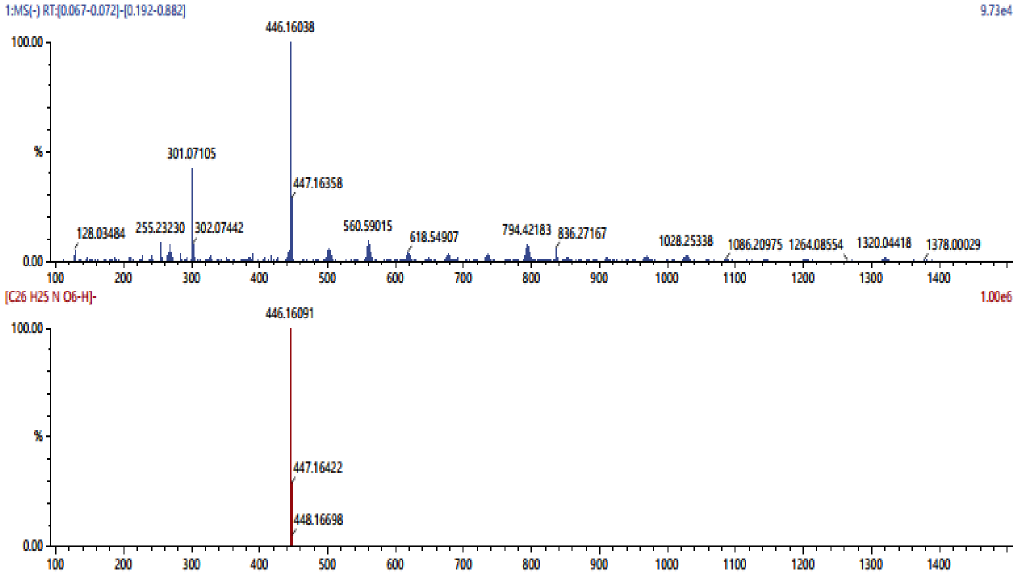

.... Page 1 of 1

HRMS of **3d**

HRMS : m/z [M-H]$^-$ calcd for. $C_{26}H_{25}NO_6$: 446.16091, found: 446.16038.

Formula Predictor Report Printed at 2020/12/15 21:31:40

Formula Predictor Result	C31 H29 N O6
Mass	510.19256
Error Margin	5 ppm
DBE Range	Not Used
Electron Ions	Both configurations
HC Ratio	Not Used
Nitrogen Rule	Not Used

#	Score	Pred. (M)	Pred. m/z	Meas. m/z	Diff. (mDa)	Formulae (M)	Ion	Diff. (ppm)	Iso Score	DBE
1	98.99	511.19949	510.19221	510.19256	0.35	C31 H29 N O6	[M-H]-	0.686	99.21	18.0

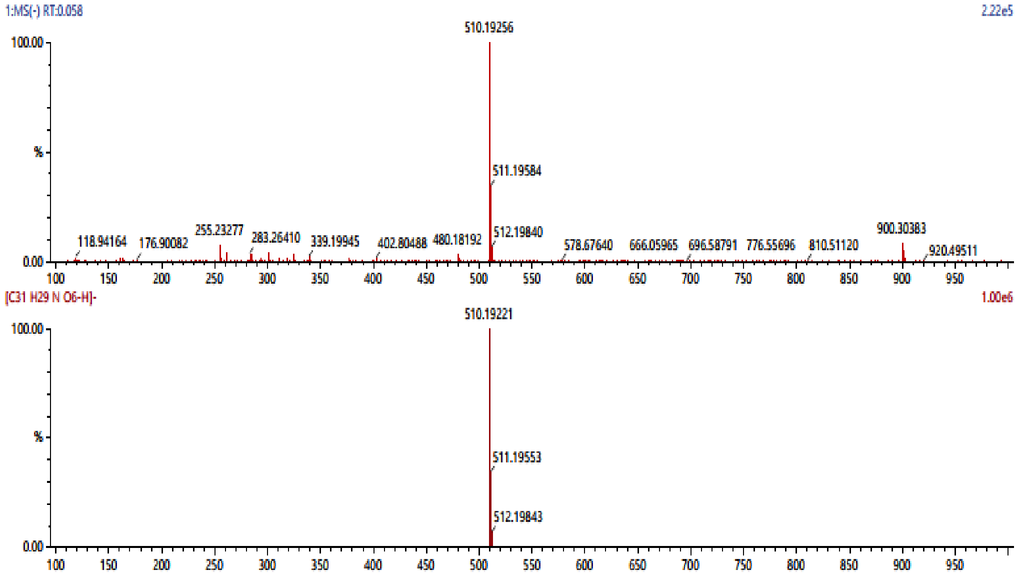

HRMS of **3e**

HRMS : m/z [M-H]$^-$ calcd for. $C_{31}H_{29}NO_6$: 510.19221, found: 510.19256.

Formula Predictor Report

Printed at 2020/12/15 21:33:09

Formula Predictor Result	C31 H29 N O6
Mass	510.19277
Error Margin	5 ppm
DBE Range	Not Used
Electron Ions	Both configurations
HC Ratio	Not Used
Nitrogen Rule	Not Used

#	Score	Pred. (M)	Pred. m/z	Meas. m/z	Diff. (mDa)	Formulae (M)	Ion	Diff. (ppm)	Iso Score	DBE
1	97.62	511.19949	510.19221	510.19277	0.56	C31 H29 N O6	[M-H]-	1.098	97.92	18.0

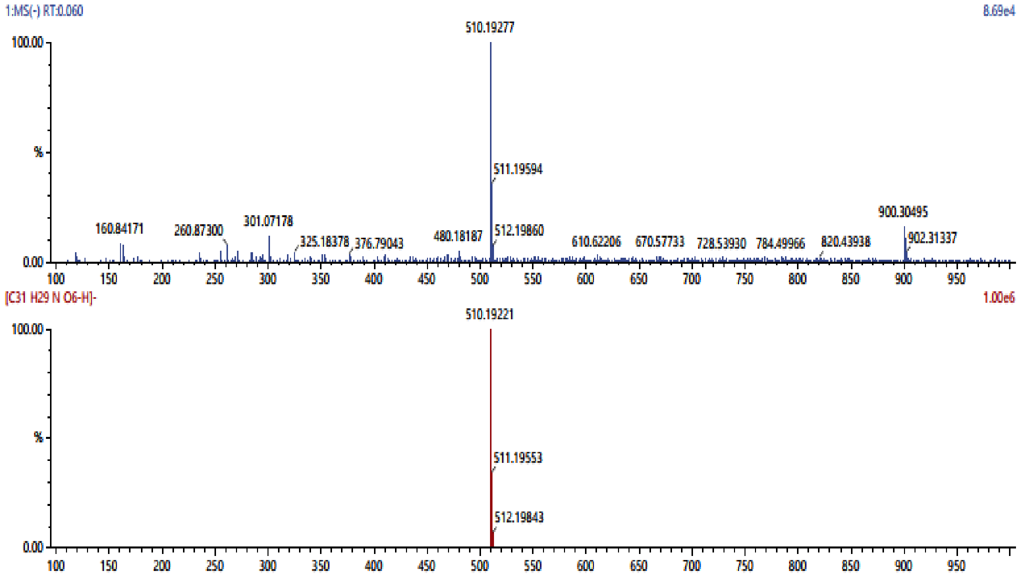

****

Page 1 of 1

HRMS of **3f**

HRMS : m/z [M-H]$^-$ calcd for. $C_{31}H_{29}NO_6$: 510.19221, found: 510.19277.

Formula Predictor Report

Printed at 2021/3/16 19:39:46

Formula Predictor Result	C30 H27 N O6
Mass	496.1757
Error Margin	10 ppm
DBE Range	Not Used
Electron Ions	Both configurations
HC Ratio	Not Used
Nitrogen Rule	Used

#	Score	Pred. (M)	Pred. m/z	Meas. m/z	Diff. (mDa)	Formulae (M)	Ion	Diff. (ppm)	Iso Score	DBE
3	85.23	497.18384	496.17656	496.17570	-0.86	C30 H27 N O6	[M-H]-	-1.733	84.58	18.0

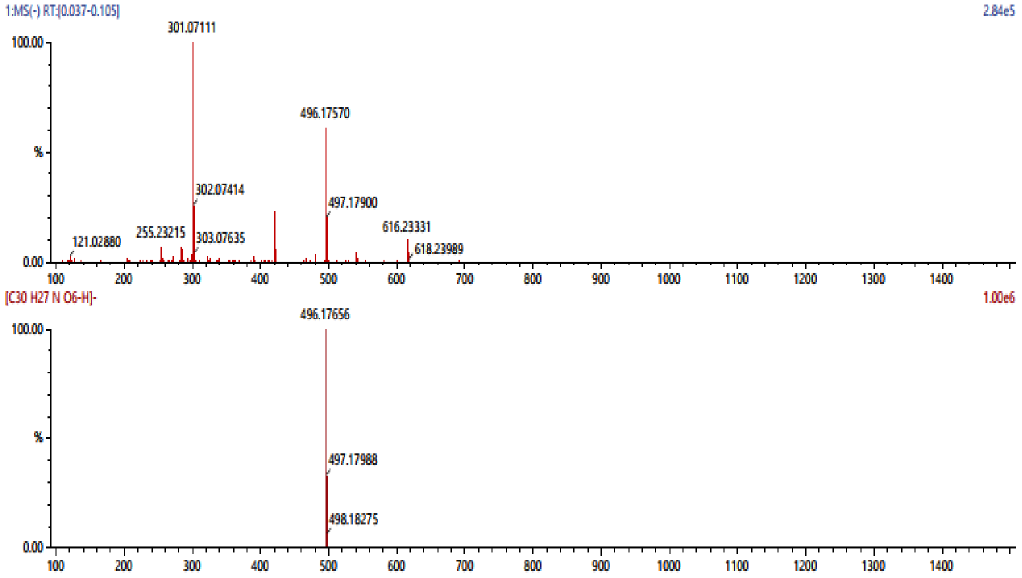

HRMS of **3g**

HRMS : m/z [M-H]$^-$ calcd for. $C_{30}H_{27}NO_6$: 496.17656, found: 496.17570.

Formula Predictor Report

Printed at 2020/12/15 21:25:29

Formula Predictor Result	C36 H29 N O6
Mass	570.19219
Error Margin	5 ppm
DBE Range	Not Used
Electron Ions	Both configurations
HC Ratio	Not Used
Nitrogen Rule	Not Used

#	Score	Pred. (M)	Pred. m/z	Meas. m/z	Diff. (mDa)	Formulae (M)	Ion	Diff. (ppm)	Iso Score	DBE
1	98.53	571.19949	570.19221	570.19219	-0.02	C36 H29 N O6	[M-H]-	-0.035	98.36	23.0

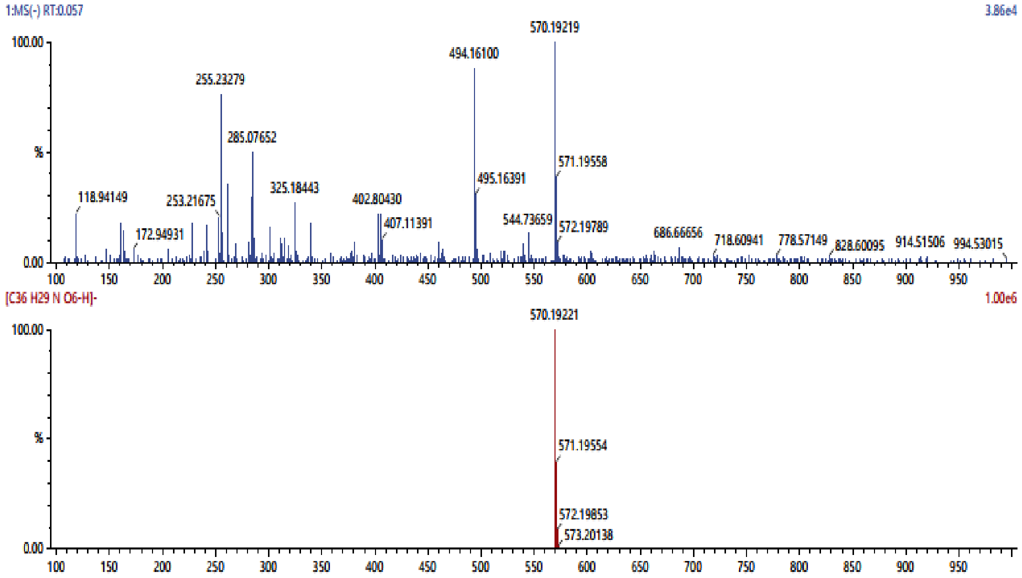

HRMS of **3h**

HRMS : m/z [M-H]$^-$ calcd for. $C_{36}H_{29}NO_6$: 570.19221, found: 570.19219.

Formula Predictor Report Printed at 2021/1/12 21:02:47

Formula Predictor Result	C29 H25 N O6
Mass	482.1604
Error Margin	5 ppm
DBE Range	Not Used
Electron Ions	Both configurations
HC Ratio	Not Used
Nitrogen Rule	Not Used

#	Score	Pred. (M)	Pred. m/z	Meas. m/z	Diff. (mDa)	Formulae (M)	Ion	Diff. (ppm)	Iso Score	DBE
1	93.99	483.16819	482.16091	482.16040	-0.51	C29 H25 N O6	[M-H]-	-1.058	93.86	18.0

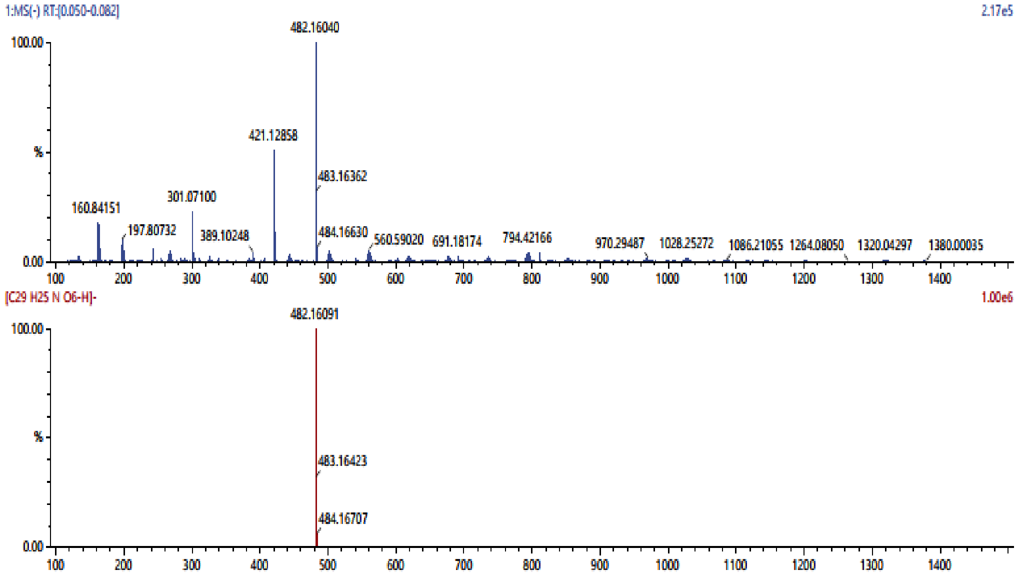

HRMS of **3i**

HRMS : m/z [M-H]$^-$ calcd for. $C_{29}H_{25}NO_6$: 482.16091, found: 482.16040.

Formula Predictor Report

Printed at 2021/3/16 19:41:05

Formula Predictor Result	C30 H27 N O6
Mass	496.17571
Error Margin	10 ppm
DBE Range	Not Used
Electron Ions	Both configurations
HC Ratio	Not Used
Nitrogen Rule	Used

#	Score	Pred. (M)	Pred. m/z	Meas. m/z	Diff. (mDa)	Formulae (M)	Ion	Diff. (ppm)	Iso Score	DBE
3	85.75	497.18384	496.17656	496.17571	-0.85	C30 H27 N O6	[M-H]-	-1.713	85.14	18.0

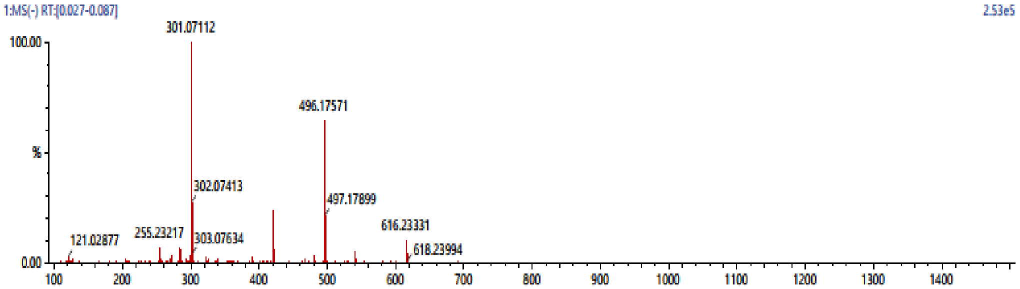

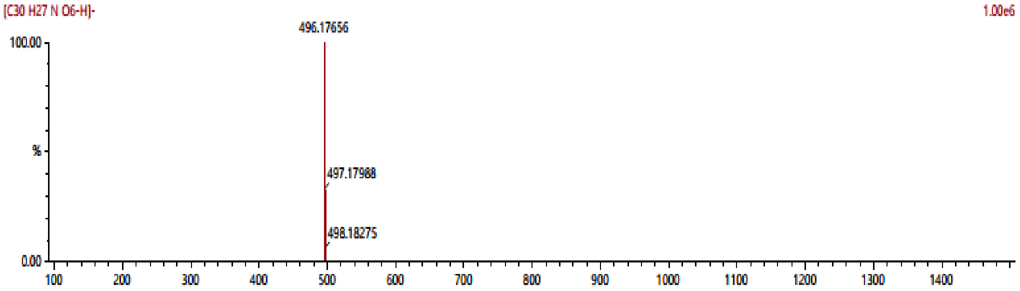

....

Page 1 of 1

HRMS of **3j**

HRMS : m/z [M-H]- calcd for. $C_{30}H_{27}NO_6$: 496.17656, found: 496.17571.

Formula Predictor Report Printed at 2020/12/15 21:34:56

Formula Predictor Result	C31 H29 N O6
Mass	510.19286
Error Margin	5 ppm
DBE Range	Not Used
Electron Ions	Both configurations
HC Ratio	Not Used
Nitrogen Rule	Not Used

#	Score	Pred. (M)	Pred. m/z	Meas. m/z	Diff. (mDa)	Formulae (M)	Ion	Diff. (ppm)	Iso Score	DBE
5	73.52	511.19949	510.19221	510.19286	0.65	C31 H29 N O6	[M-H]-	1.274	71.25	18.0

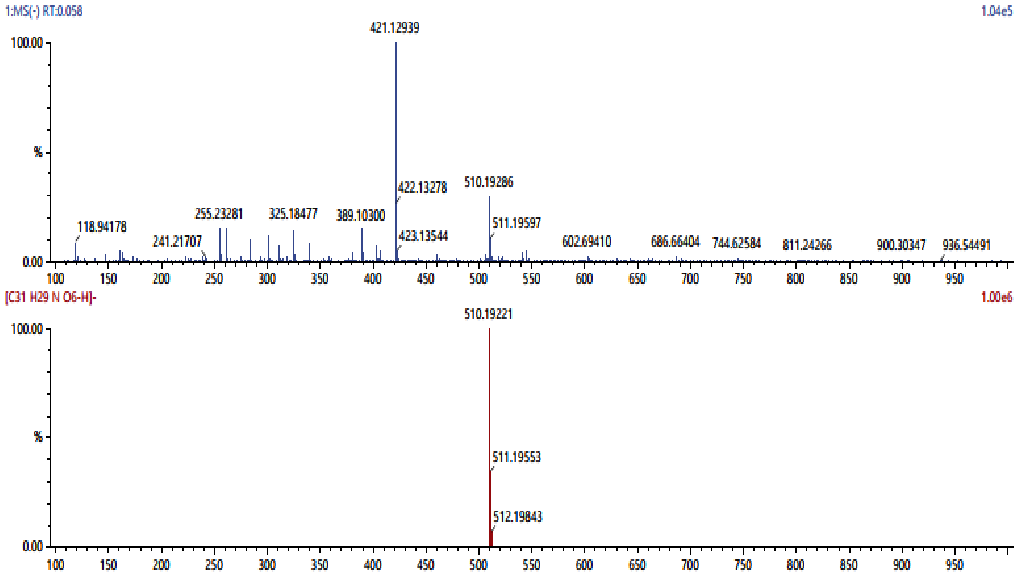

HRMS of **3k**

HRMS : m/z [M-H]+ calcd for. $C_{31}H_{29}NO_6$: 510.19221, found: 510.19286.

E. Antioxidant Activities Research

DPPH Antioxidant Activity Assay

Stable and convenient free radical 2,2 - diphenyl-1 - picrylhydrazyl (DPPH) method was used to determine the free radical scavenging activity of hesperetin and its derivatives in this study. The experimental samples were diluted with ethanol into different concentrations of sample solution, and then the accurate amount of 0.5 mL sample solution and 2.5 mL 60 μM DPPH solution were dissolved in ethanol. The mixed solution was oscillated vigorously in the dark for 1 hour, and the absorbance of the re-action solution was measured by UV spectrophotometer at 517 nm. VC as positive con-trol in this experiment. The expression formula of DPPH scavenging activity is as fol-lows:

DPPH scavenging activity (%) = $(A_C-A_S)/A_C \times 100$ (1)

Where A_C is the absorbance of DPPH solution as the negative control, and A_S is the absorbance of 0.5 mL sample in a 2.5 mL DPPH solution. All samples were analyzed in triplicate, and the results are expressed as mean±standard deviation. The scavenging activity was expressed as the 50% inhibitory concentration (IC_{50}), which was the sample concentration at which 50% of DPPH radicals were scavenged after incubation.

ABTS Antioxidant Activity Assay

The free radical scavenging ability of hesperetin and its derivatives was evaluated by stable and convenient ABTS method. The experimental samples were diluted with ethanol in turn into different concentrations of sample solutions, and then accurate amount and configured with 7 mM ABTS solution and 2.45 mM potassium persulfate solution with deionized water to produce ABTS radicals at room temperature in dark environment for 16 h. The absorbance of the accurate volume of ABTS solution diluted with ethanol to 734 nm was 0.70±0.05. The diluted ABTS solution (2 mL) was mixed with 100μL of the experimental sample, and the mixture was kept in dark at room temperature for 6 min. The absorbance of the mixed solution was measured by UV spectrophotometer at 734 nm. VC was used as the positive control in this experiment. The expression formula of ABTS scavenging activity is as follows:

$$\text{ABTS scavenging activity (\%)} = (A_C - A_S)/A_C \times 100 \quad (2)$$

where A_C is the absorbance of the ABTS solution as the control and A_S is the absorbance of the ABTS solution after adding the sample. The IC_{50} was calculated from the graph of scavenging percentage against concentration using IBM SPSS Statistics 25.0. The results are expressed as mean±standard deviation of three experiments.

F. Anti-tumor activity assays

Study on the cytotoxicity of Hesperetin and Hesperetin Derivatives to MCF-7, HeLa and HepG2 cell lines by the methyl thiazolyl tetrazolium (MTT) assays. First of all, MCF-7 cell lines were seeded into a 96-well cell culture plate. The seed concentration is about 1×10^4/hole, then, the cell lines were cultured at 37 °C under 5%

CO_2 for 24 h. After that, the concentration of Hesperetin and Hesperetin Derivatives (0, 0.01, 0.1, 1, 10, 50 μM) were added in these wells respectively. After incubated for another 48 h at the same conditions, the MTT solutions (10 μL, 0.5 mg/mL) were added to these wells. Then, cells were incubated for 4h. The medium was removed and 100 μL dimethyl sulfoxide was added to dissolve the formazan crystals. The OD_{570} was measured by an ELx800 microplate reader (Bio-Tek, Winooski, VT, USA) with subtraction of background absorbance. The cytotoxicity of Hesperetin and Hesperetin Derivatives were expressed as an IC_{50}, defined as the concentration causing a 50% reduction of cell growth compared with untreated cells. The cell viability was calculated using following equation.

$$\text{Cell inhibition ratio (\%)} = (A_C - A_S)/A_C \times 100 \quad (3)$$

Where A_C is the absorbance of control, and A_S is the absorbance of dyes. All samples were analyzed in triplicate, and the results are expressed as mean±standard deviation. The cytotoxicity of hesperetin and hesperetin derivatives (3**a**-3**k**) was expressed as an IC_{50}, defined as the concentration causing a 50% reduction of cell growth compared with untreated cells.

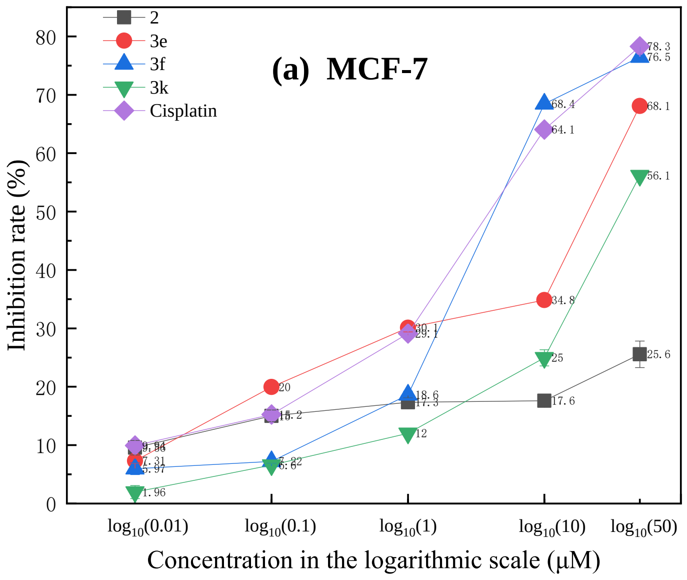

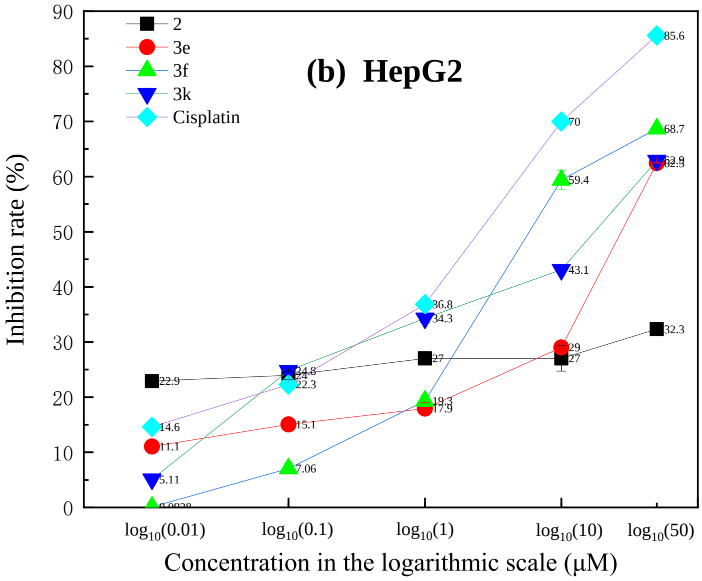

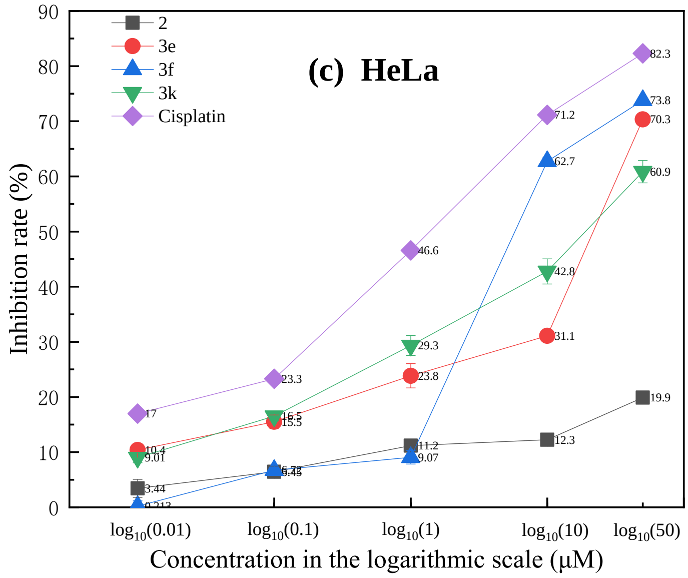

Figure S1. Cytotoxicity evaluation of hesperetin 2 and derivatives 3**e**, 3**f**, 3**k** and Cisplatin on MCF-7 (a); HepG2 (b); HeLa (c). (The treatment time used in cytotoxicity studies is 48 h)

G. ^{1}H and ^{13}C NMR Spectra of Compounds

^{1}H NMR and ^{13}C NMR of product (**2**)

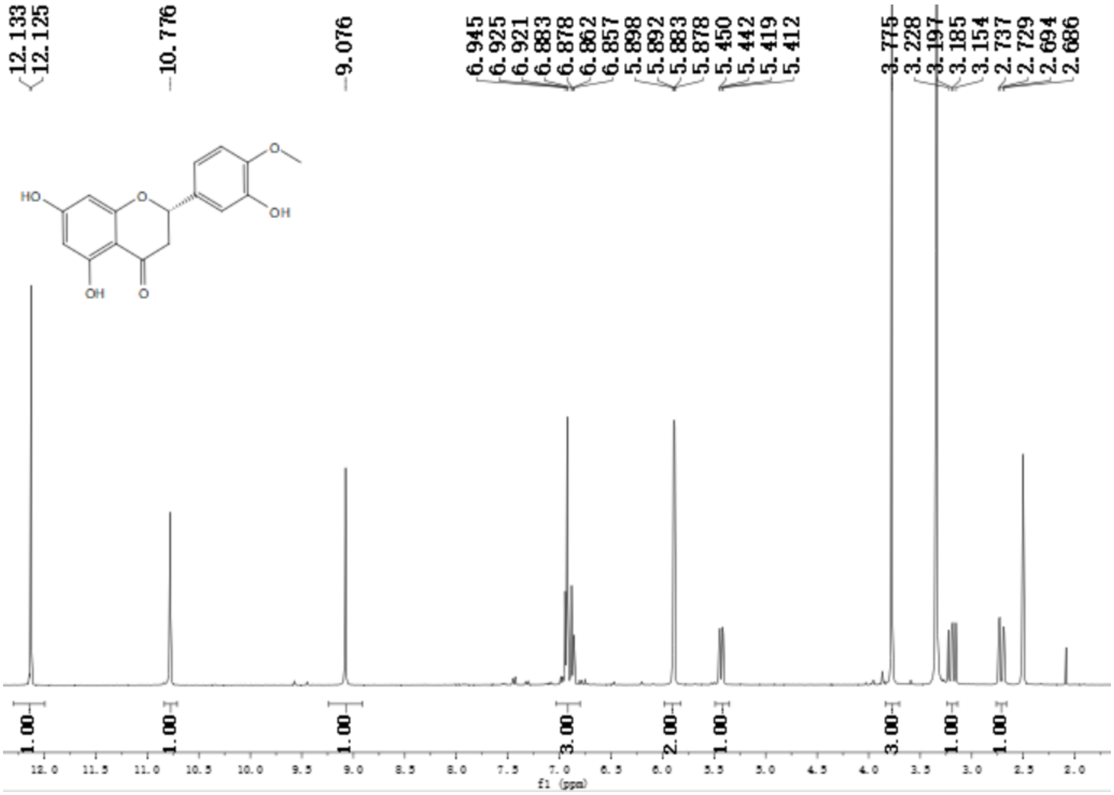

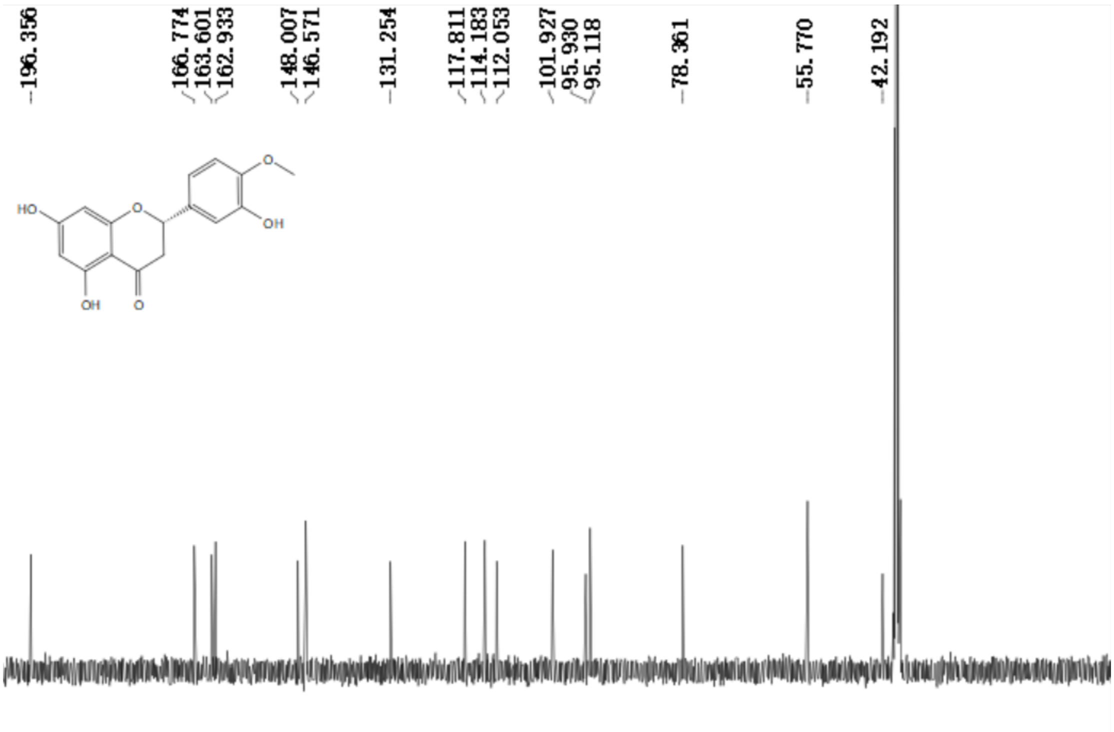

^{1}H NMR and ^{13}C NMR of product (**3a**)

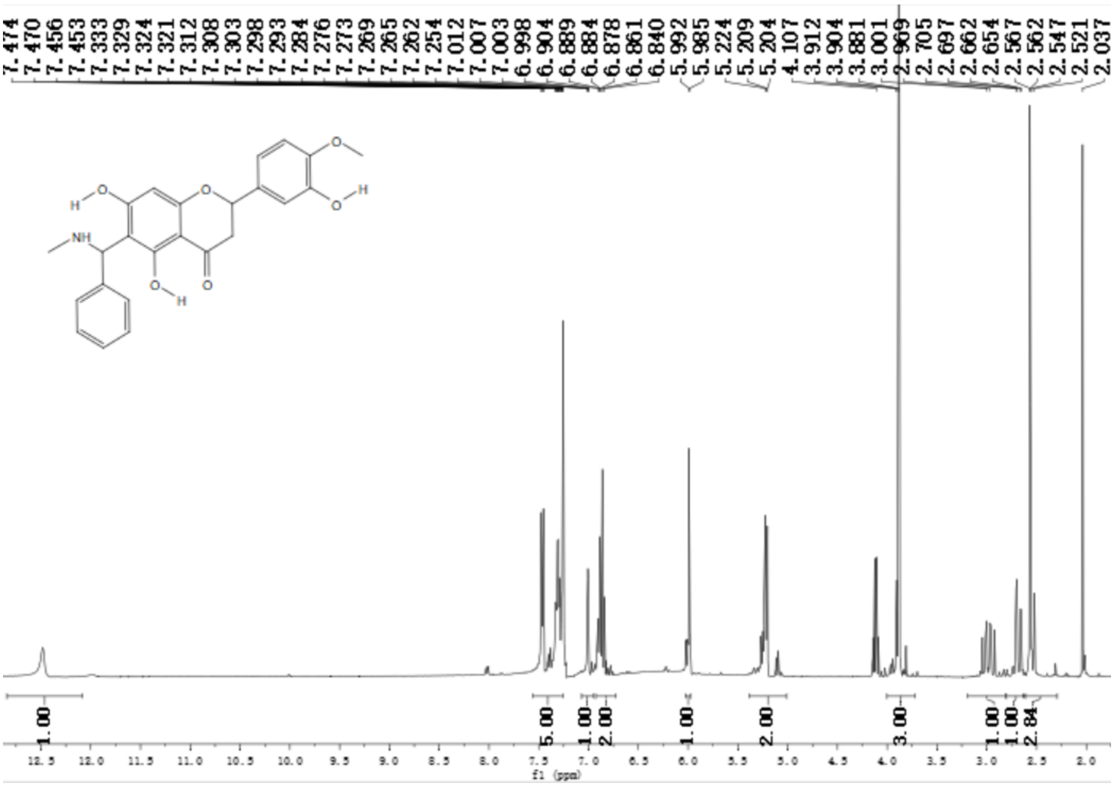

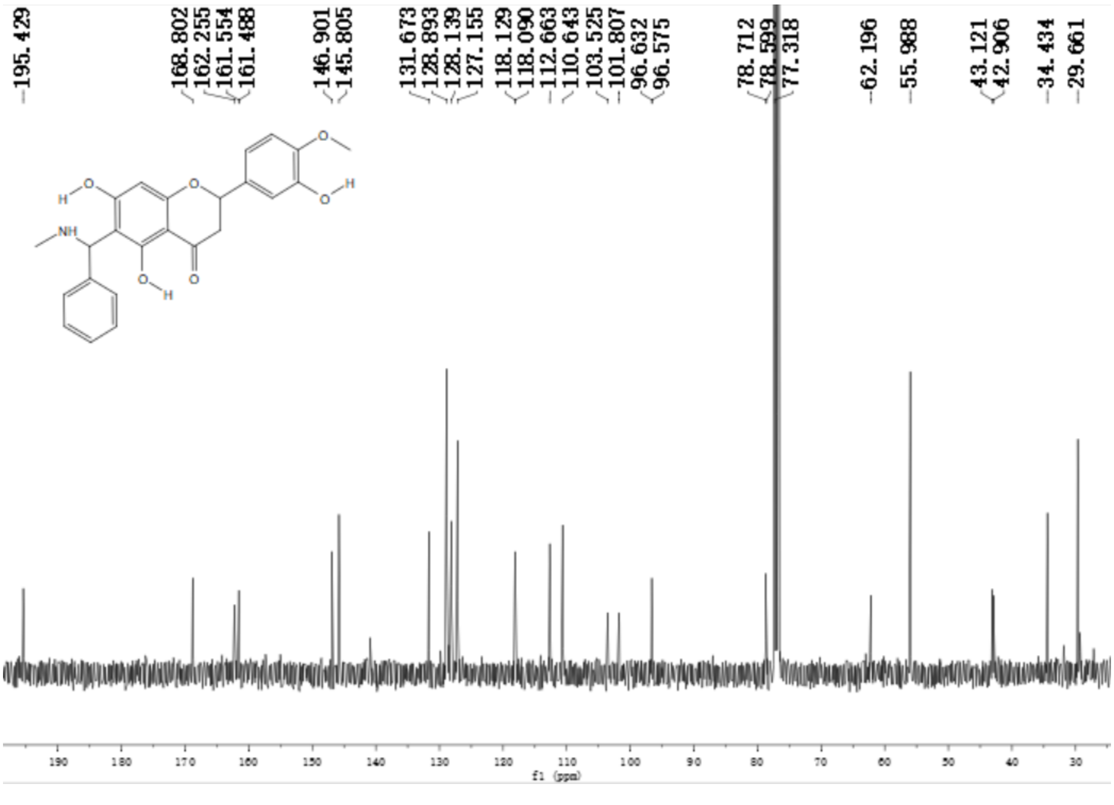

^{1}H NMR and ^{13}C NMR of product (**3b**)

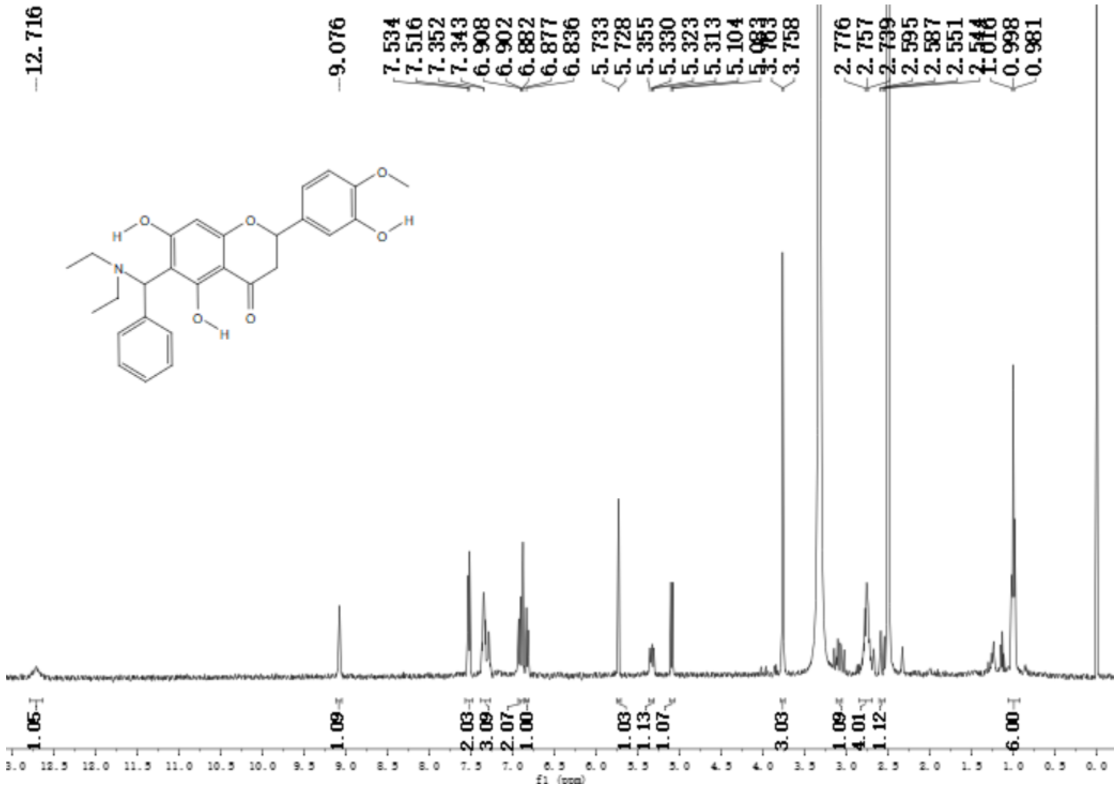

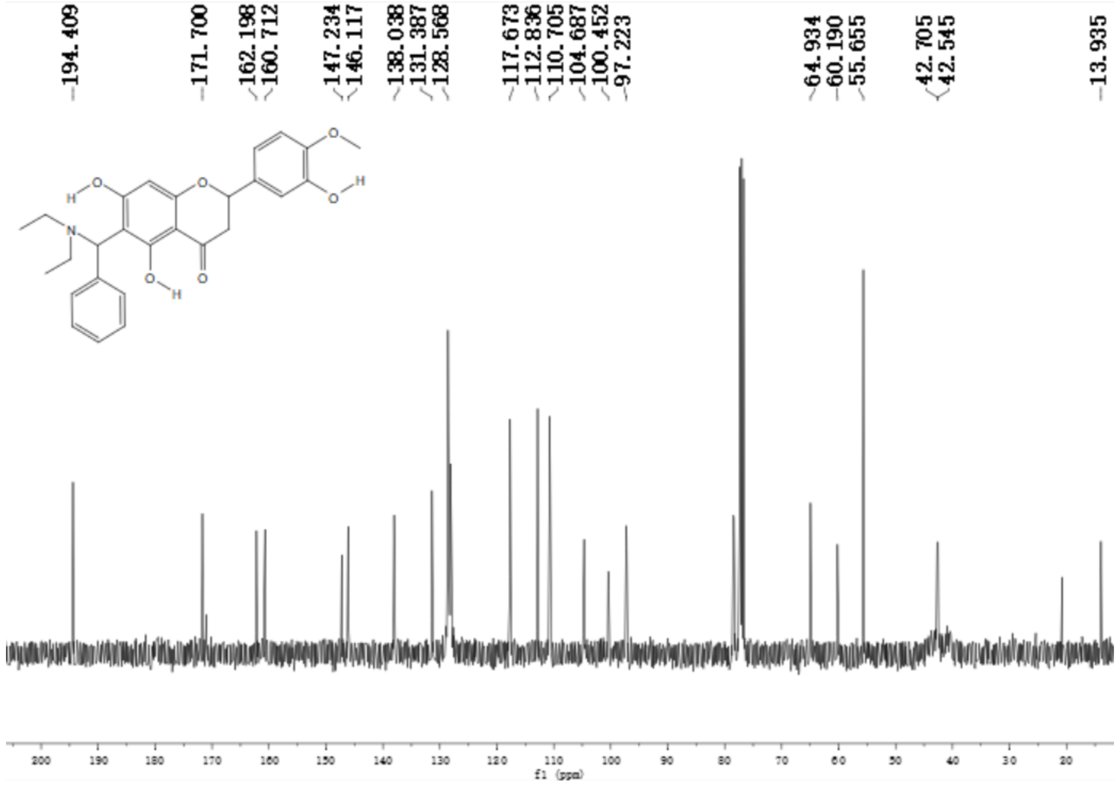

^{1}H NMR and ^{13}C NMR of product (**3c**)

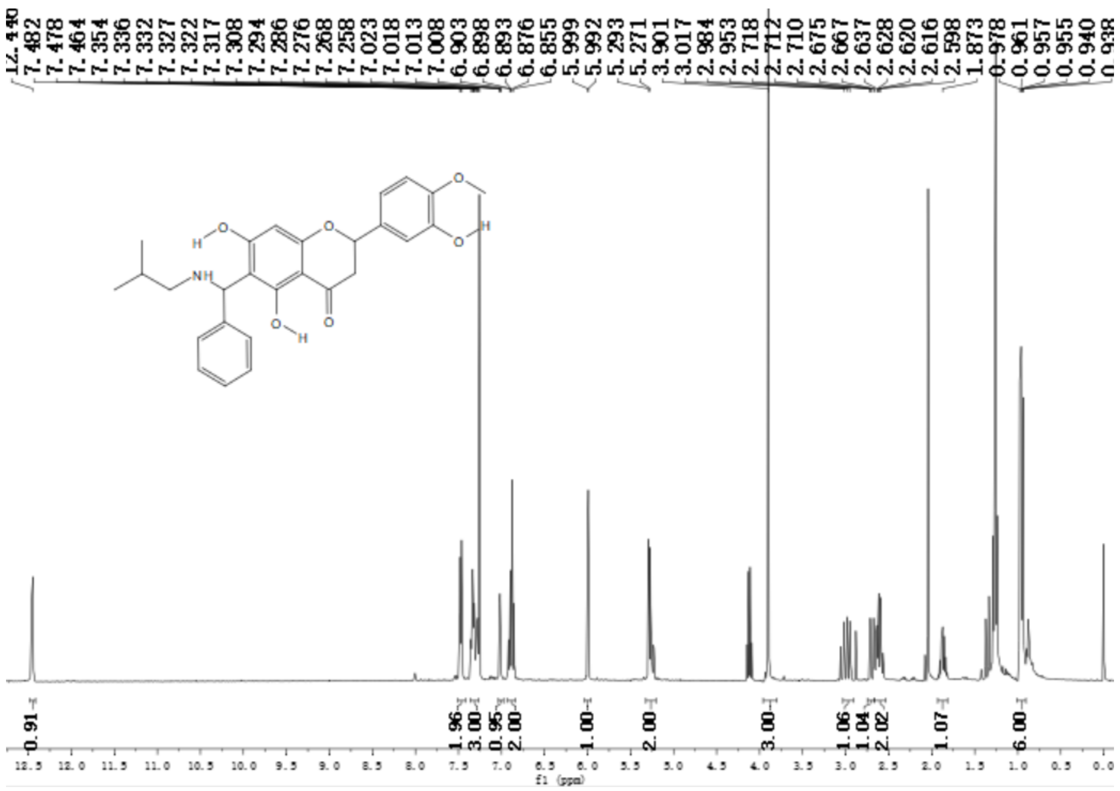

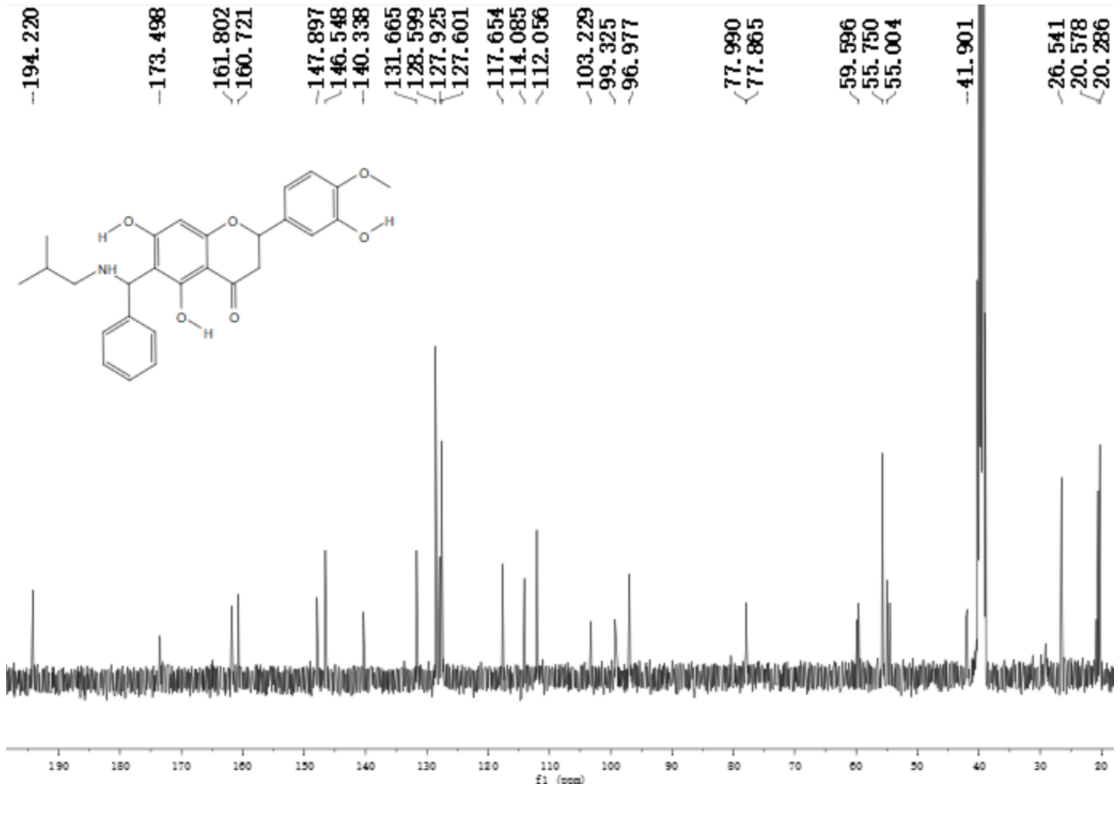

^{1}H NMR and ^{13}C NMR of product (**3d**)

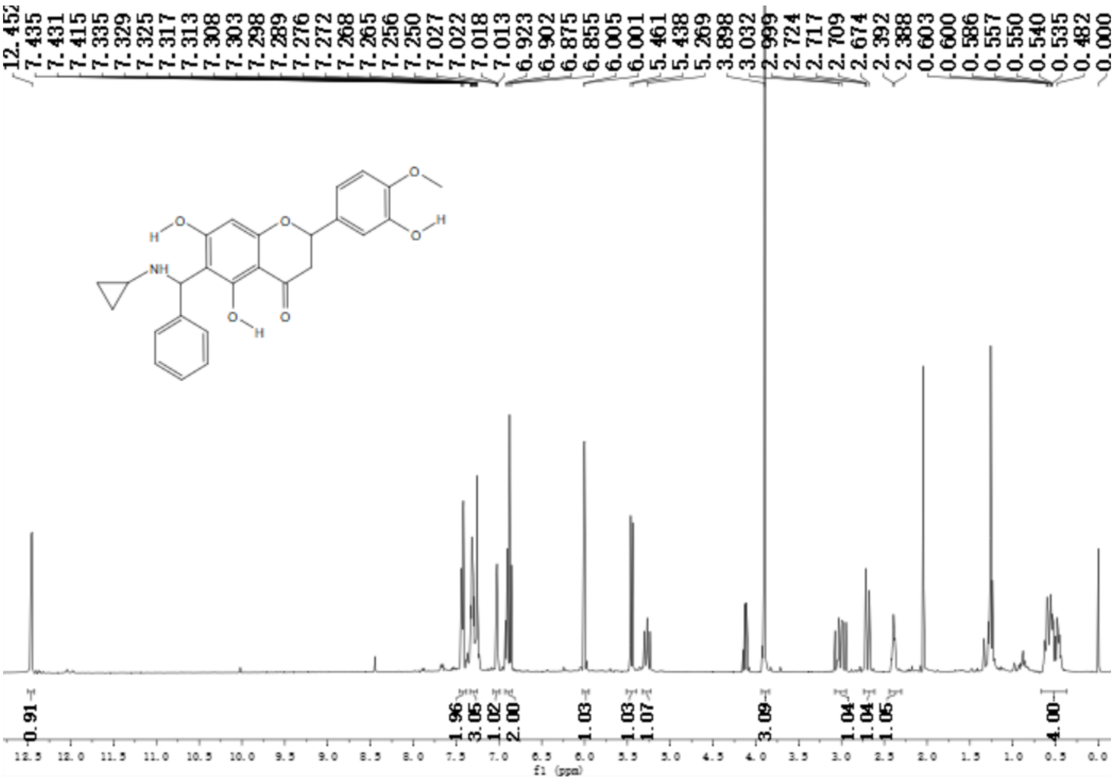

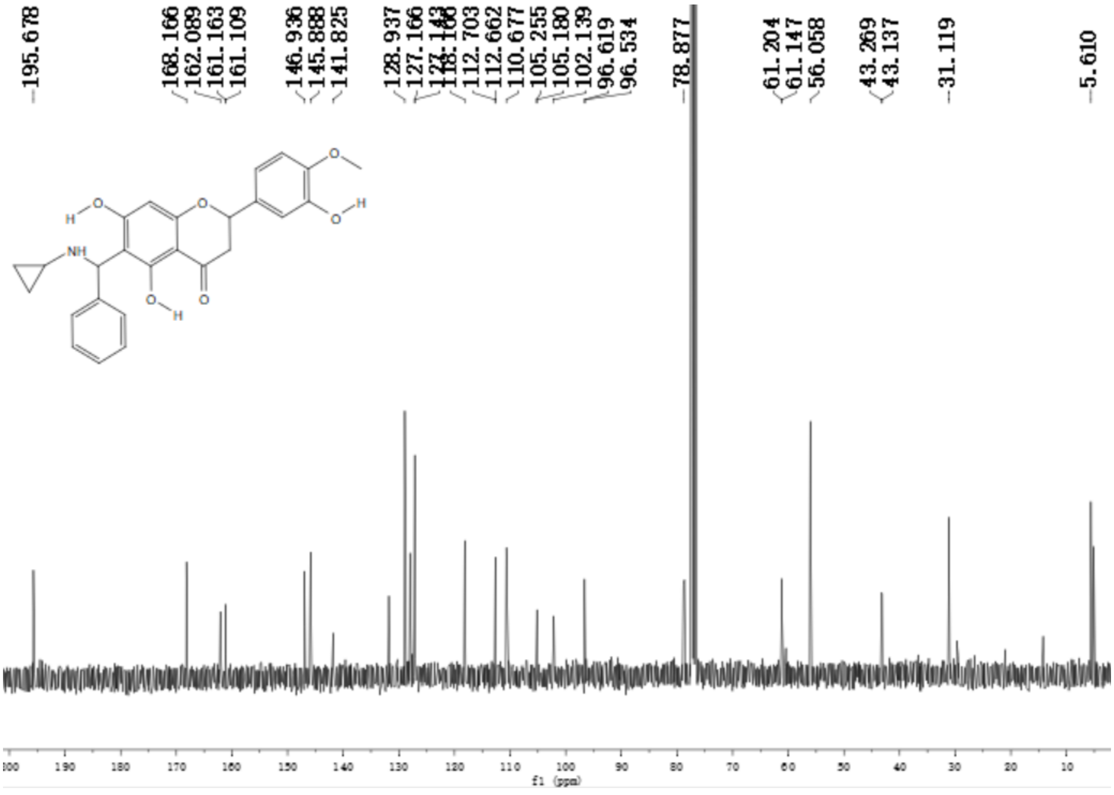

^{1}H NMR and ^{13}C NMR of product (**3e**)

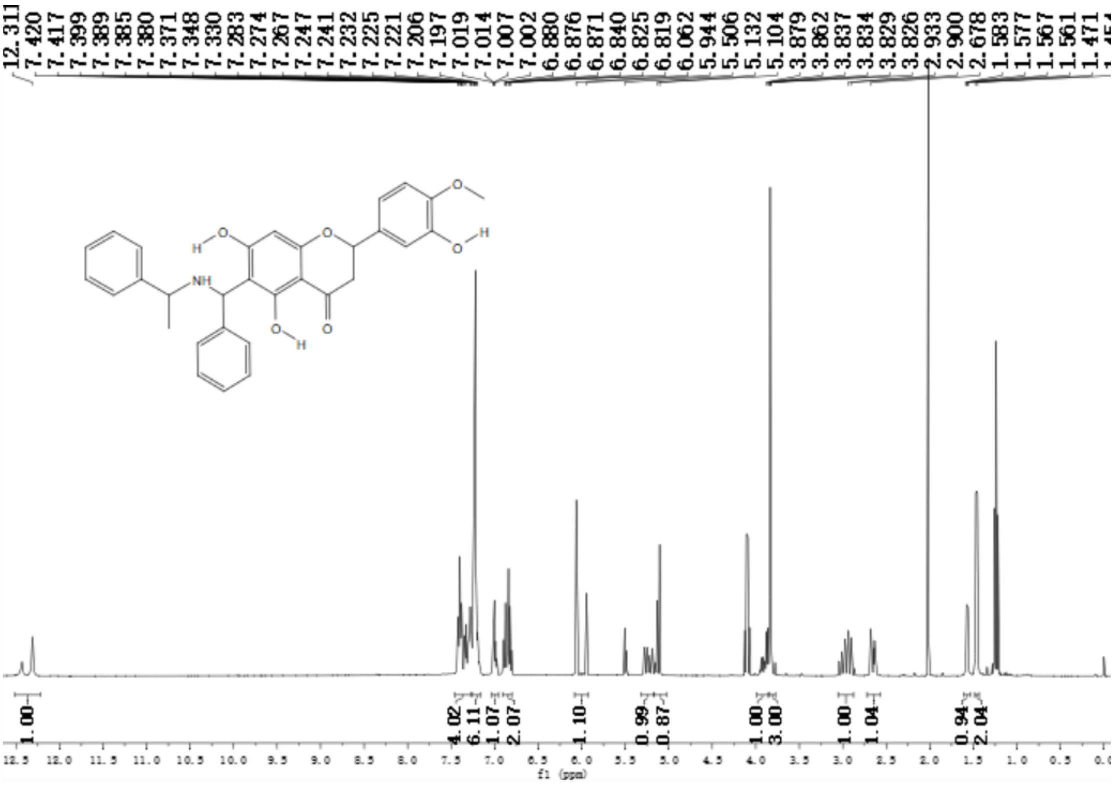

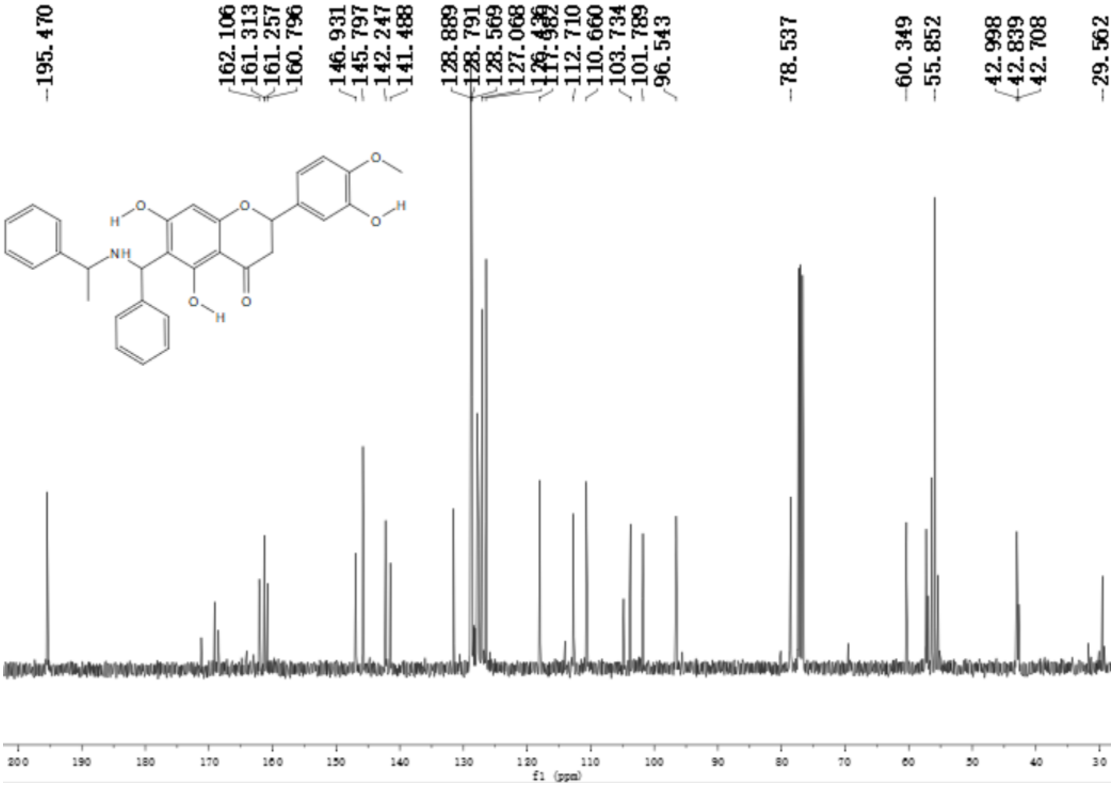

^{1}H NMR and ^{13}C NMR of product (**3f**)

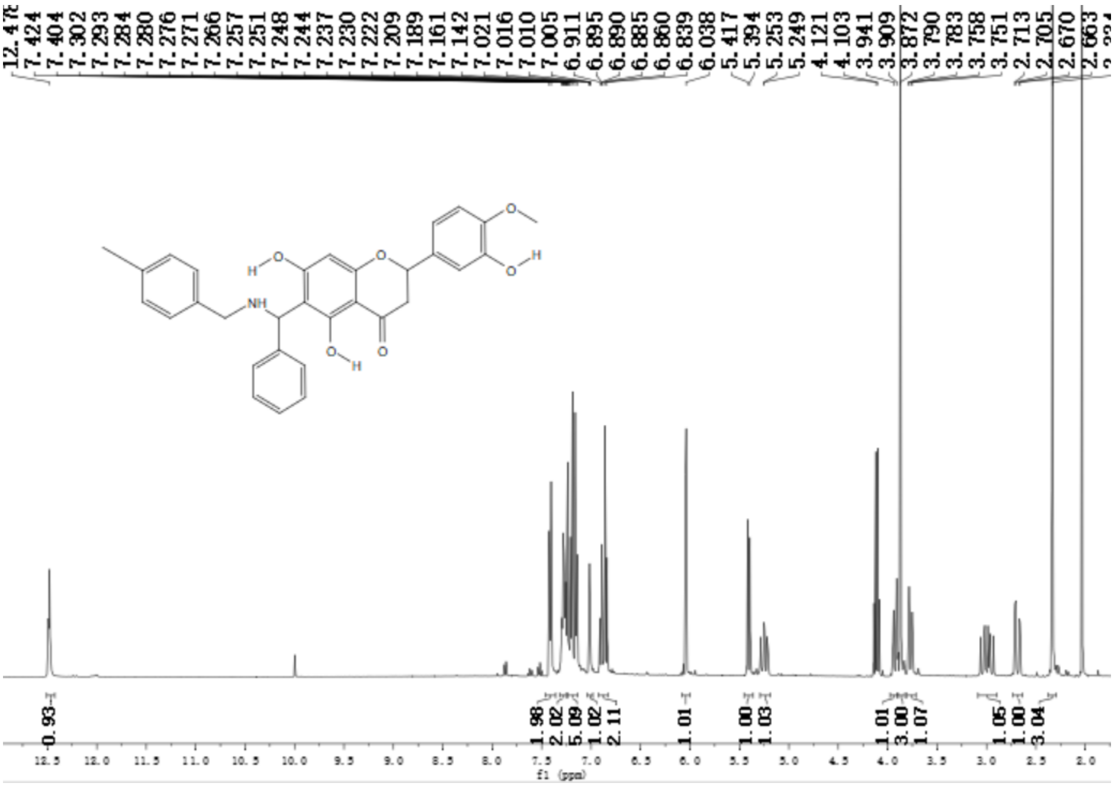

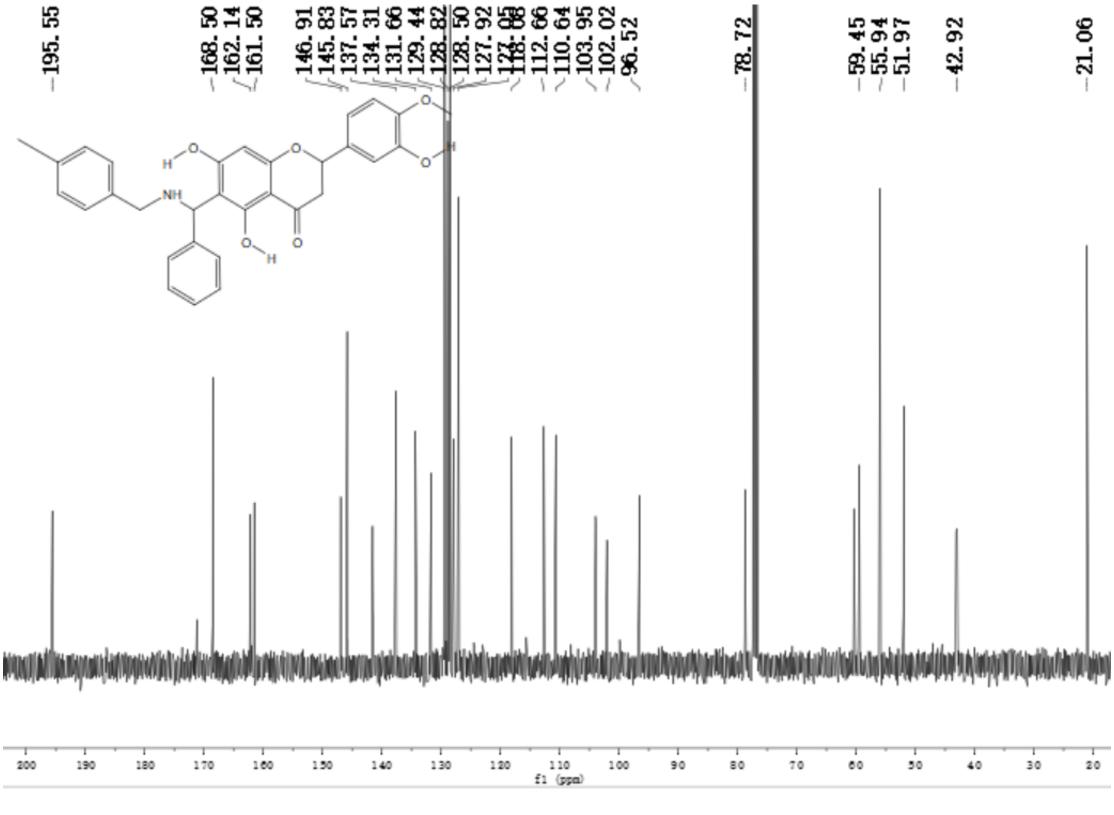

^{1}H NMR and ^{13}C NMR of product (**3g**)

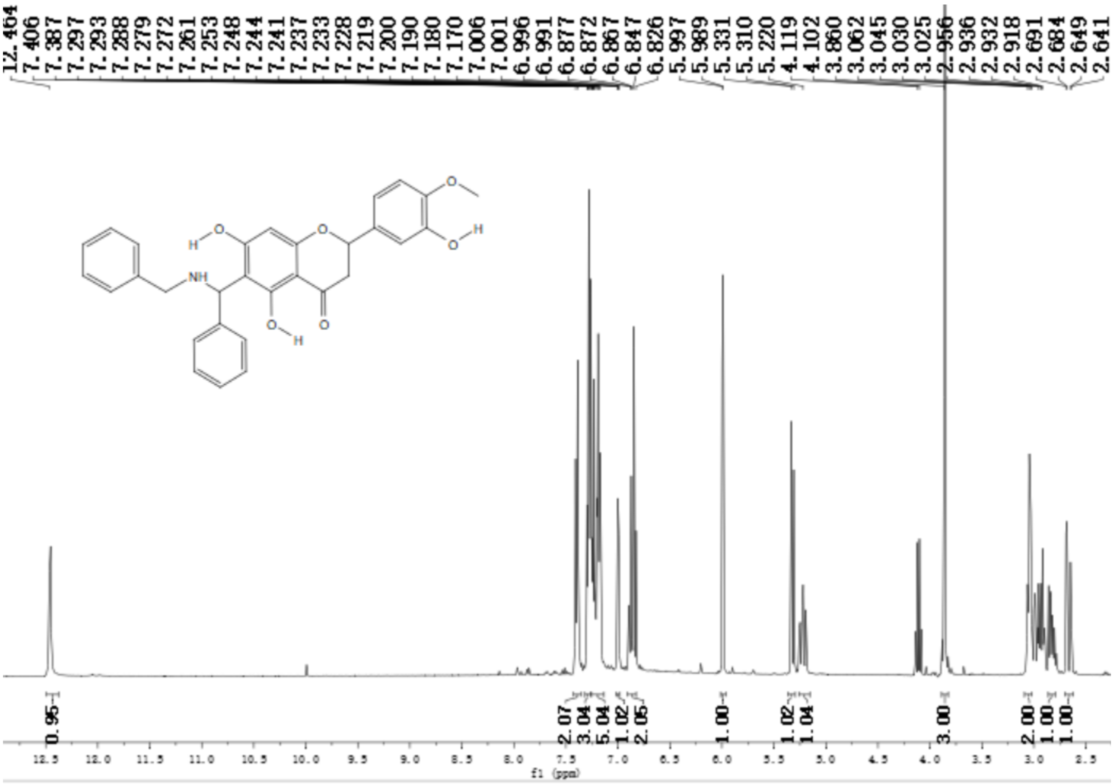

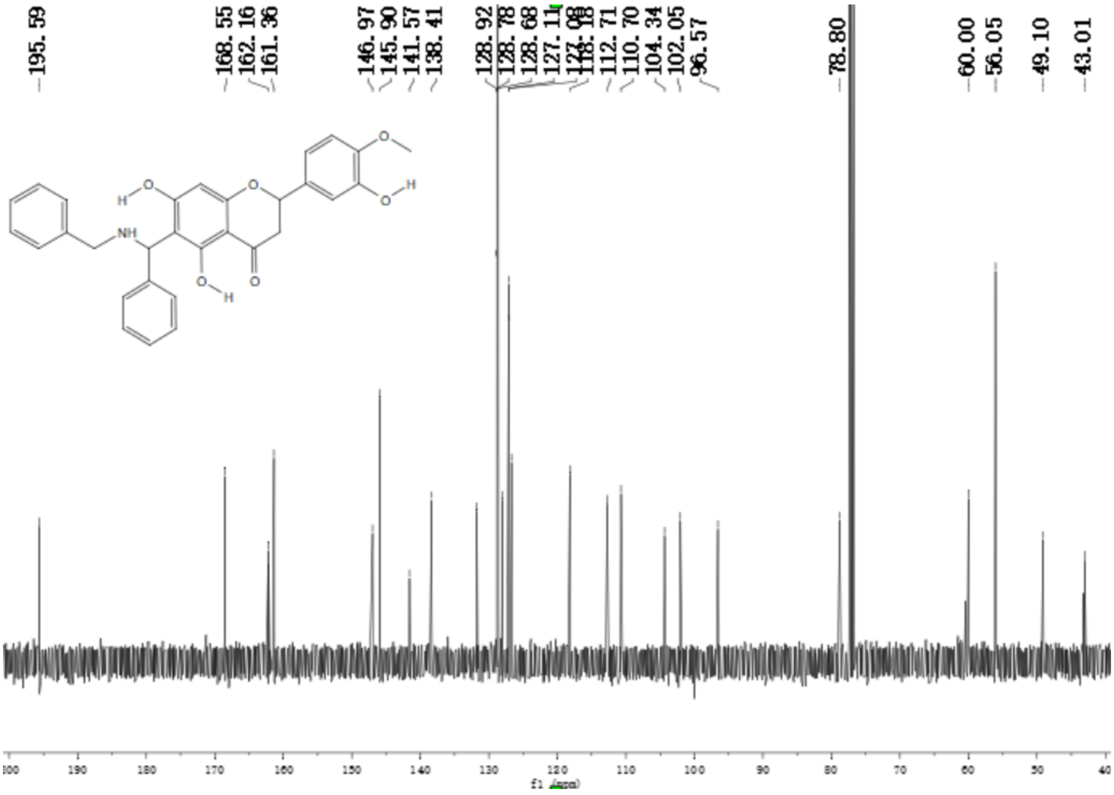

^{1}H NMR and ^{13}C NMR of product (**3h**)

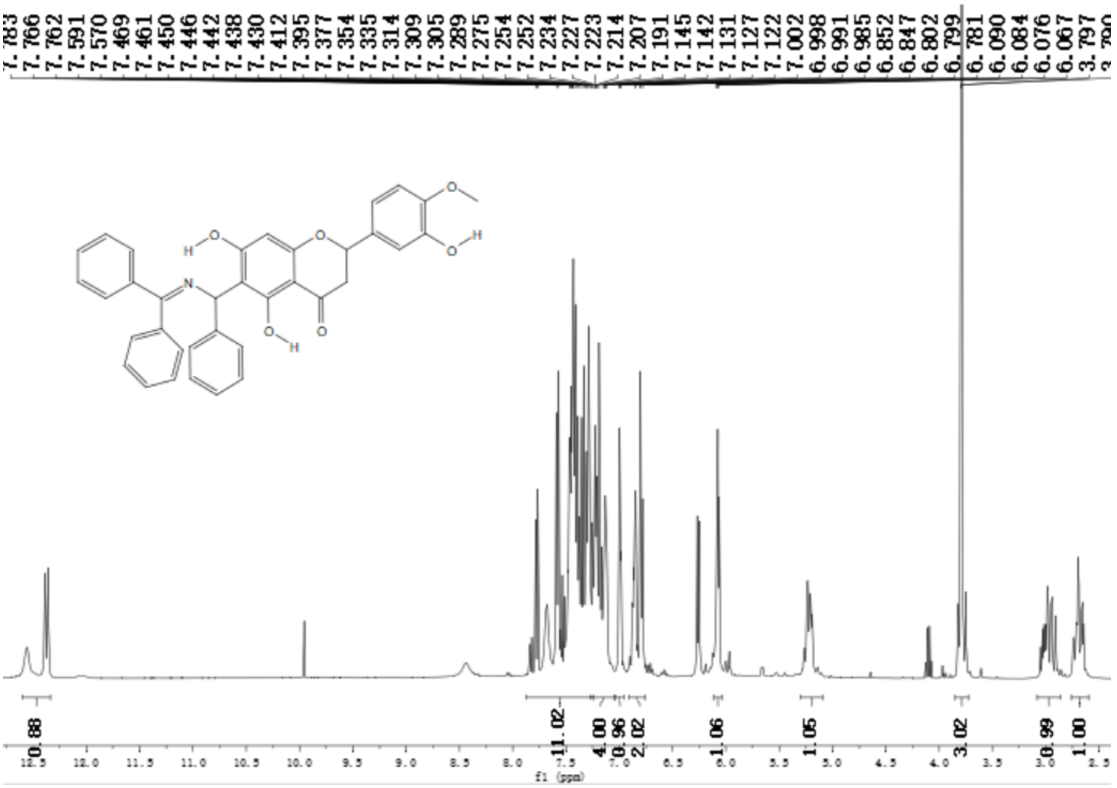

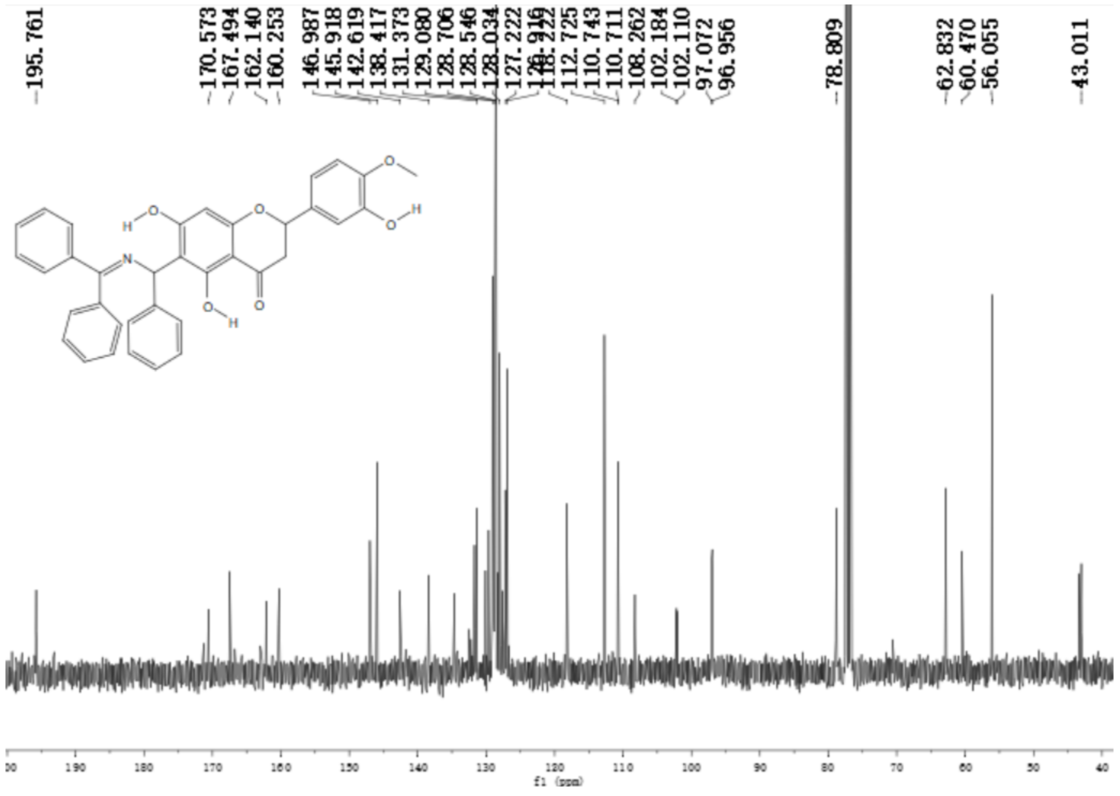

^{1}H NMR and ^{13}C NMR of product (**3i**)

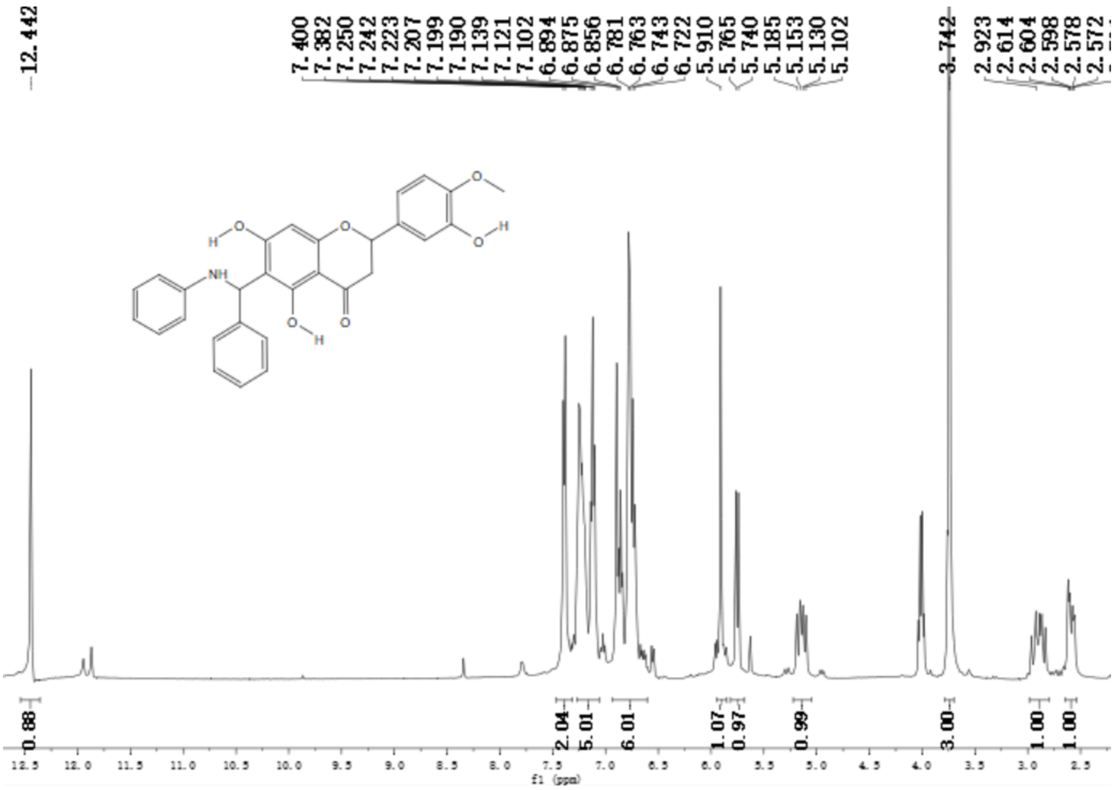

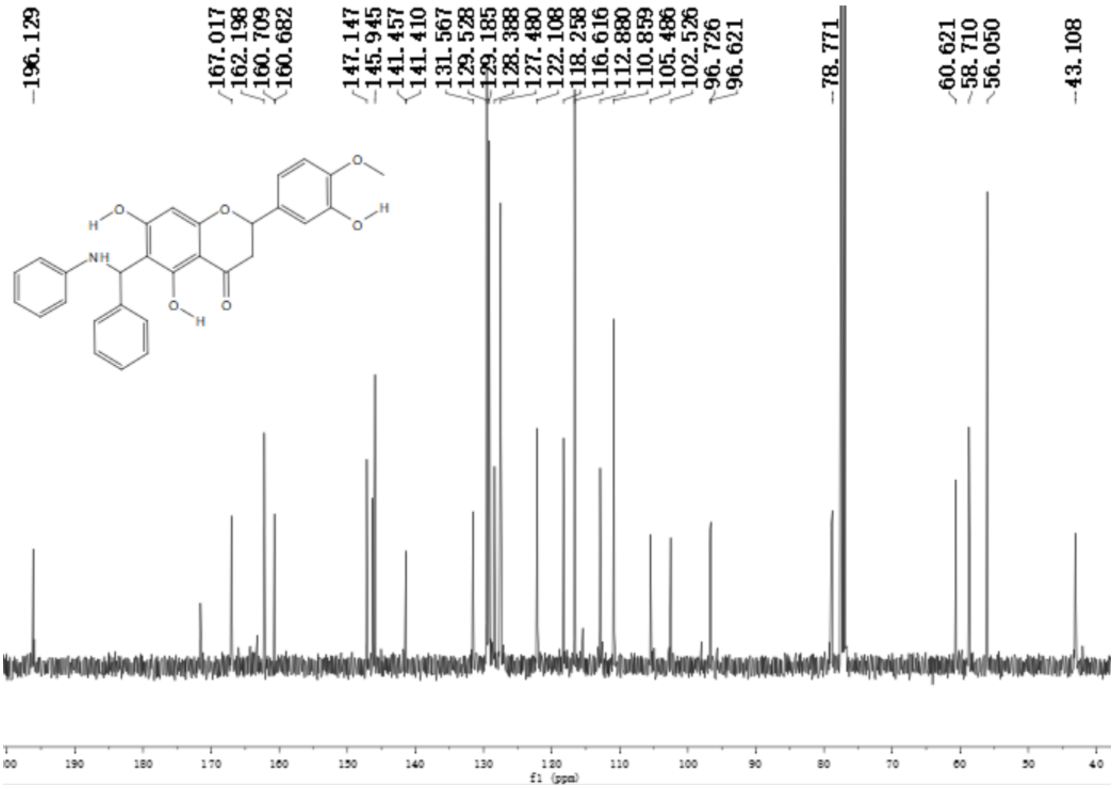

^{1}H NMR and ^{13}C NMR of product (**3j**)

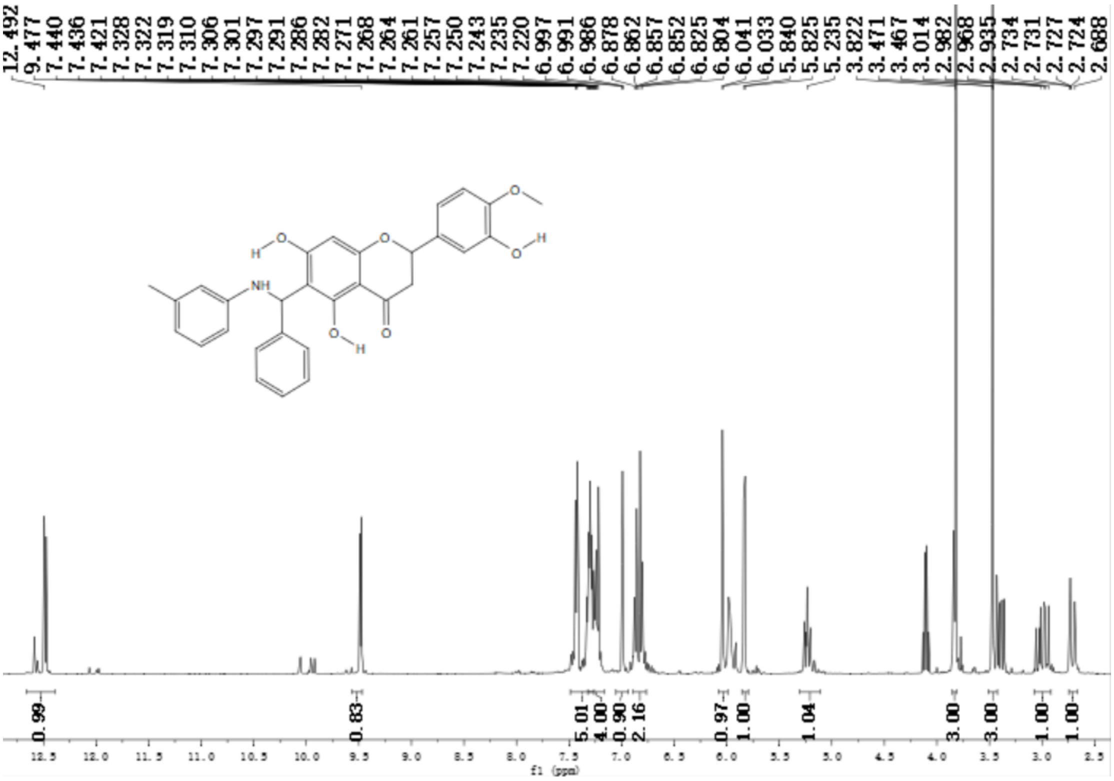

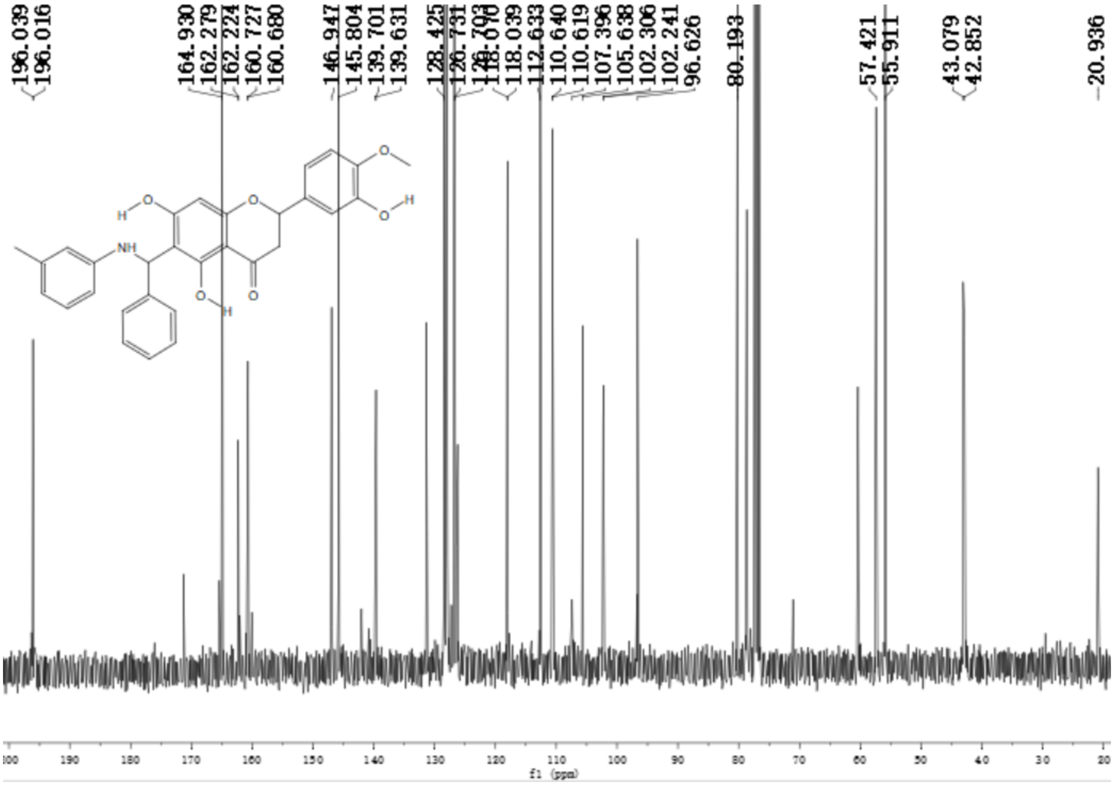

^{1}H NMR and ^{13}C NMR of product (**3k**)

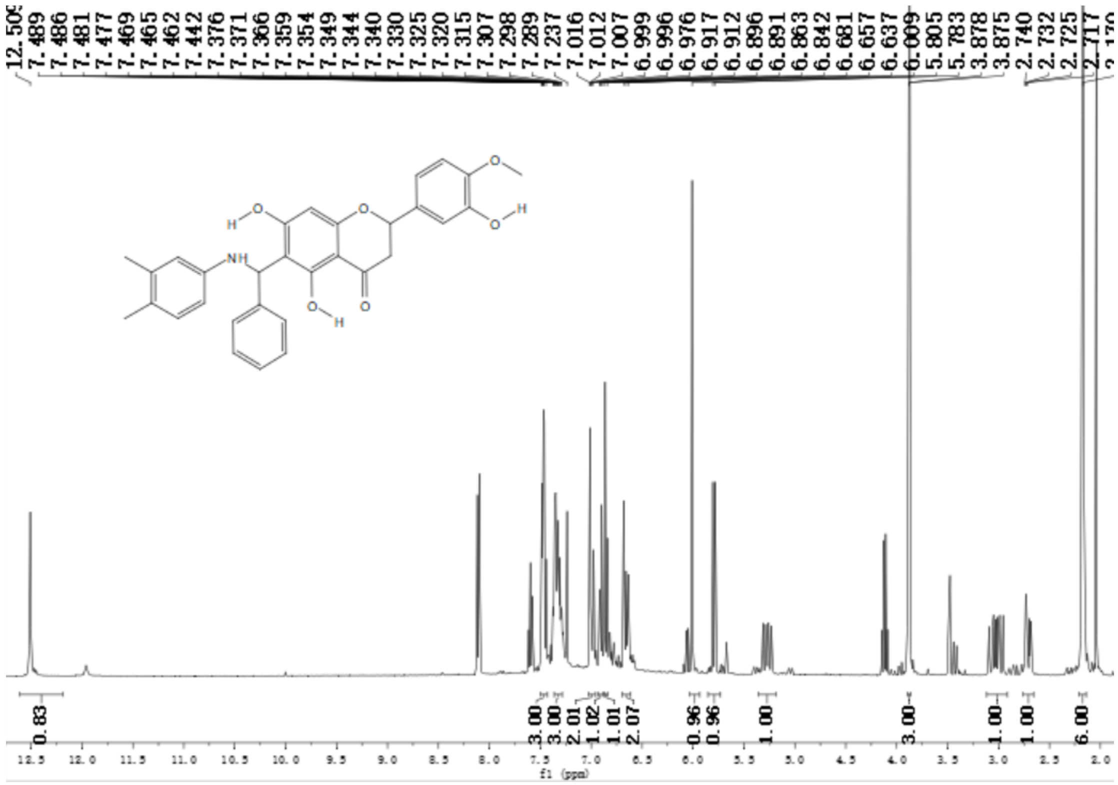

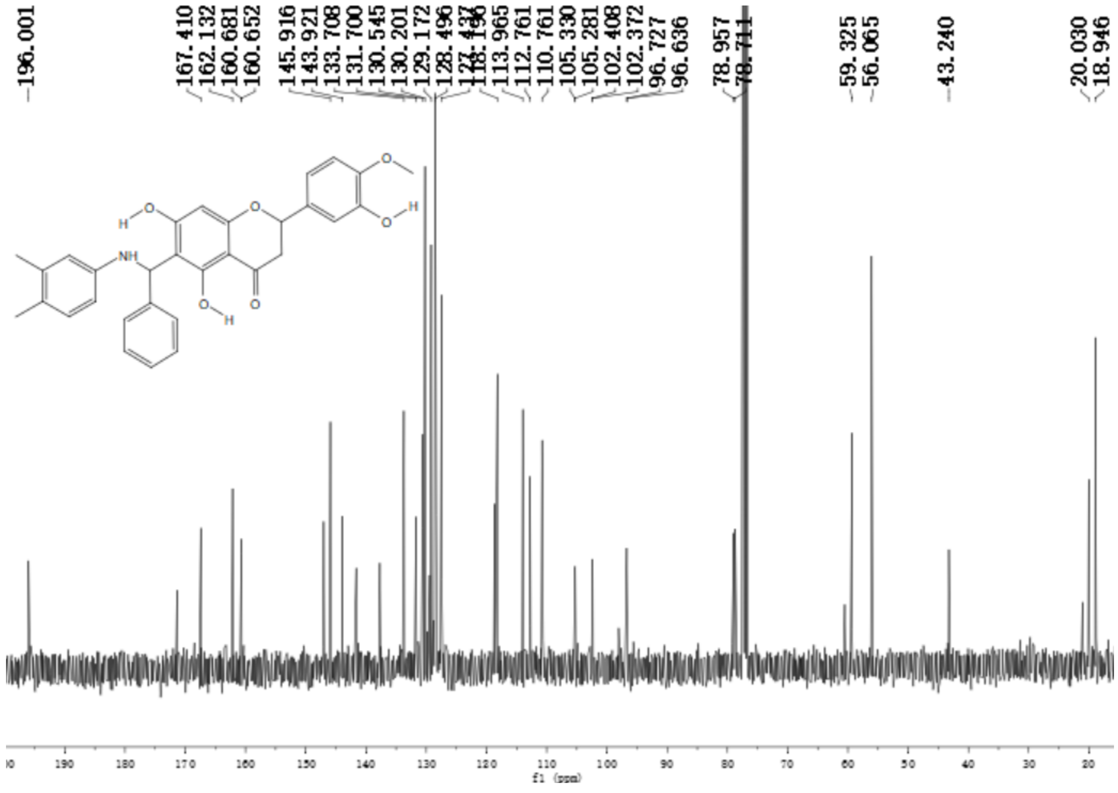

Supplement: Supplementary file 1 [file molecules-27-00879-s001.zip › molecules-1547543-supplementary.pdf]
